# Preventive and Therapeutic Effects of Chinese Herbal Compounds against Hepatocellular Carcinoma

**DOI:** 10.3390/molecules21020142

**Published:** 2016-01-27

**Authors:** Bing Hu, Hong-Mei An, Shuang-Shuang Wang, Jin-Jun Chen, Ling Xu

**Affiliations:** 1 Department of Oncology and Institute of Traditional Chinese Medicine in Oncology, Longhua Hospital, Shanghai University of Traditional Chinese Medicine, Shanghai 200032, China; wss885489@163.com (S.-S.W.); xulq67@aliyun.com (L.X.); 2 Department of Science & Technology, Longhua Hospital, Shanghai University of Traditional Chinese Medicine, Shanghai 202032, China; lhsoatp@163.com; 3 Department of Plastic & Reconstructive Surgery, Shanghai Key Laboratory of Tissue Engineering, The Ninth People’s Hospital, School of Medicine, Shanghai Jiaotong University, Shanghai 200011, China; chenjj30003000@aliyun.com

**Keywords:** hepatocellular carcinoma, Chinese herbal compound, phytopharmacology, prevention, treatment

## Abstract

Traditional Chinese Medicines, unique biomedical and pharmaceutical resources, have been widely used for hepatocellular carcinoma (HCC) prevention and treatment. Accumulated Chinese herb-derived compounds with significant anti-cancer effects against HCC have been identified. Chinese herbal compounds are effective in preventing carcinogenesis, inhibiting cell proliferation, arresting cell cycle, inducing apoptosis, autophagy, cell senescence and anoikis, inhibiting epithelial-mesenchymal transition, metastasis and angiogenesis, regulating immune function, reversing drug resistance and enhancing the effects of chemotherapy in HCC. This paper comprehensively reviews these compounds and their effects on HCC. Finally, the perspectives and rational application of herbal compounds for HCC management are discussed.

## 1. Introduction

Despite advances in diagnosis and treatment, hepatocarcinoma remains the sixth most common malignancy and the third principal cause of cancer deaths worldwide [1], underscoring the need to develop novel approaches for liver cancer control. Traditional Chinese Medicines (TCMs) play a positive role in the management of hepatocarcinoma [2,3]. TCMs are effective in alleviating clinical symptoms, improving quality of life and immune function, preventing recurrence and metastasis, delaying tumor progression, and prolonging survival of hepatocarcinoma patients [4]. Accumulated Chinese herbal compounds have been identified and demonstrated anti-cancer effects against hepatocellular carcinoma (HCC). Chinese herbal compounds represent an important medical and pharmaceutical resource for the development of new treatments for hepatocarcinoma. These compounds and their effects on HCC are comprehensively reviewed in present work.

## 2. Prevention of Hepatocarcinogenesis

**Figure 1:**
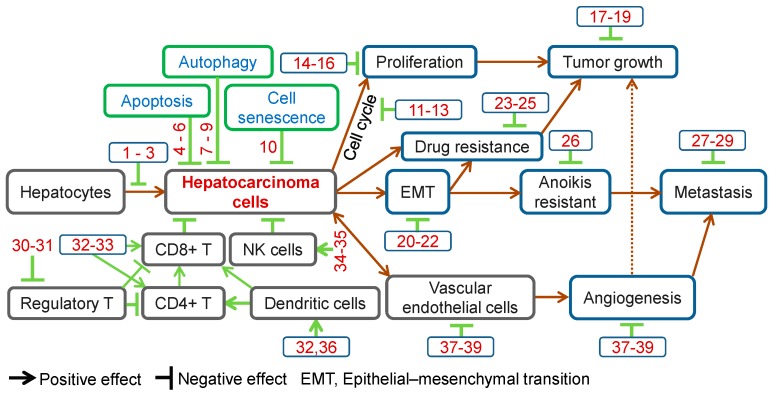
Anti-cancer effects of herbal compounds against hepatocarcinoma. 1, Ursolic acid; 2, Penta-acetyl geniposide; 3, Curcumin; 4, Matrine; 5, Solamargine; 6, Ponicidin; 7, Tetrandrine; 8, Baicalein; 9, Bufalin; 10, Ganoderiol F; 11, Rhein; 12, Oridonin; 13, Curcumol; 14, Salvianolic acid B; 15, Steroidal saponins; 16, Davidiin; 17, β-Elemene; 18, Ardipusilloside-I; 19, Raddeanin A; 20, Tanshinone IIA; 21, Cordycepin; 22, Huaier polysaccharides; 23, Astragaloside II; 24, Oroxylin A; 25, Tetramethylpyrazine; 26, Arecoline; 27, Artemisinin; 28, Resveratrol; 29, Isofraxidin; 30, Astragalus polysaccharides; 31, *Radix Glycyrrhizae* polysaccharides; 32, *Lycium barbarum* polysaccharide; 33, Polysaccharides from *Artemisia annua* L.; 34, Gastrodin; 35, Shikonin; 36, Gekko sulfated polysaccharide-protein complex; 37, Gekko-sulfated glycopeptide; 38, Pedicularioside G; 39, Vitexin compound **1**.

TCMs have long been used for disease prevention, including liver cancer. Increasing numbers of Chinese herbal compounds have been isolated and demonstrated preventive effects against hepatocarcinogenesis. Ursolic acid is a natural triterpenoid widely found in Chinese herbs, such as *Gardenia jasminoides* Ellis (Zhi-Zi), *Prunella vulgaris* (Xia-Ku-Cao) and *Hedyotis diffusa* Willd. (Bai-Hua-She-She-Cao). Ursolic acid is effective in preventing diethylnitrosamine (DEN)-induced oxidative stress and hepatocarcinogenesis [5,6] (Figure 1, Table 1).

**Table 1:** Herbal compounds that inhibit hepatocarcinogenesis.

Compounds	Herbs	Effects	Targets/Molecular Events	Ref.
Ursolic acid	*Gardenia jasminoides* Ellis (Zhi-Zi), *Prunella vulgaris* (Xia-Ku-Cao), *Hedyotis diffusa* Willd. (Bai-Hua-She-She-Cao), *etc.*	↓ DEN induced hepatocarcinogenesis	↓ Oxidative stress	[5,6]
Penta-acetyl geniposide	*Gardenia jasminoides* Ellis (Zhi-Zi)	↓ AFB1 induced hepatocarcinogenesis	↓ GGT foci	[7]
Curcumin	*Curcuma kwangsiensis* (Yu-Jin or Er-Zhu), *C. phaeocaulis* (Yu-Jin or Er-Zhu), *C. wenyujin* (Yu-Jin or Er-Zhu), *C. longa* (Yu-Jin or Jiang-Huang), *etc.*	↓ DEN induced hepatocarcinogenesis	↓ p21(ras), PCNA and CDC2	[8]
Berberine	*Coptis chinensis* Franch. (Huang-Lian), *Phellodendron chinense* Schnied. (Huang-Bai)	↓ DEN-plus-PB induced hepatocyte proliferation	↓ iNOS, cytochrome P450, CYP2E1 and CYP1A2	[9]
Saikosaponin-d	*Bupleurum chinense* (Chai-Hu)	↓ DEN induced hepatocarcinogenesis	↓ COX-2 and C/EBPβ	[10]
Gomisin A	the fruits of *Schisandra chinensis* or *Schisandra sphenanthera* (Wu-Wei-Zi)	↓ 3′-MeDAB induced hepatocarcinogenesis	Unknown	[11]
Tea polyphenols and tea pigments	Tea	↓ DEN induced hepatocarcinogenesis	↑ p21WAF1 and Bax, ↓ Bcl-2	[12]
Astragalosides, Astragalus polysaccharide and salvianolic acids	*Astragalus membranaceous* (Huang-Qi), *Salvia miltiorrhiza Bunge* (Dan-shen)	↓ DEN induced hepatocarcinogenesis	↓ GST-P and α-SMA	[13]

↓ Inhibit or down-regulate, ↑ up-regulate; DEN, diethylnitrosamine; AFB1, aflatoxin B1; PB, phenobarbital; 3′-MeDAB, 3′-methyl-4-dimethylaminoazobenzene.

Pentaacetyl geniposide, a component of *G. jasminoides* Ellis (Zhi-Zi), protects rats from aflatoxin B1 (AFB1)-induced hepatocarcinogenesis [7] (Figure 1). Curcumin, a common component present in *Curcuma kwangsiensis* (Yu-Jin or Er-Zhu), *C. phaeocaulis* (Yu-Jin or Er-Zhu), *C. wenyujin* (Yu-Jin or Er-Zhu) or *C. longa* (Yu-Jin or Jiang-Huang), is effective in preventing DEN-induced hepatocarcinogenesis accompanied by down-regulation of p21(ras), PCNA and CDC2 [8] (Figure 1). Berberine, a component of *Coptis chinensis* Franch. (Huang-Lian) or *Phellodendron chinense* Schnied. (Huang-Bai), inhibits hepatocyte proliferation induced by DEN and phenobarbital (PB) [9] (Table 1).

Saikosaponin-d, a compound isolated from *Bupleurum chinense* (Chai-Hu) inhibits DEN-induced hepatocarcinogenesis via down-regulation of COX-2 and CCAAT/enhancer binding protein β (C/EBPβ) [10]. The fruits of *Schisandra chinensis* or *S. sphenanthera* (Wu-Wei-Zi) inhibit mutagenicity and hepatocarcinogenesis induced by AFB1 [14,15]. Gomisin A, a component of these fruits, inhibits 3′-methyl-4-dimethylaminoazobenzene-induced hepatocarcinogenesis [11]. Tea polyphenols and tea pigments up-regulate p21WAF1 and Bax, and down-regulate Bcl-2 to inhibit DEN-induced hepatocarcinogenesis [12] (Table 1).

The compound *Astragalus* and *Salvia miltiorrhiza* extract, a herbal component formula composed of astragalosides, *Astragalus* polysaccharide and salvianolic acids, has demonstrated efficacy in preventing DEN-induced hepatocarcinoma in a dose-dependent manner, accompanied by down-regulation of glutathione S-transferase placental type (GST-P) and α-SMA [13] (Table 1).

## 3. Inhibition of Cell Proliferation

Cancer is characterized by uncontrolled cell proliferation and tumor growth. Inhibition of cell proliferation and tumor growth is one of the primary goals of cancer therapy. Some herbal compounds are effective in inhibiting HCC cell/tumor growth.

Salvianolic acid B, isolated from *S. miltiorrhiza* Bunge (Dan-Shen), inhibits proliferation in hepatoma cells [16]. Steroidal saponins, derived from the rhizomes of *Dioscorea bulbifera* (Huang-Du or Huang-Yao-Zi), inhibit cell proliferation in HCC cells [17]. Davidiin, extracted from *Polygonum capitatum* (Tou-Hua-Liao), inhibits cell proliferation and tumor growth in HCC by targeting EZH2 [18] (Figure 1, Table 2).

β-Elemene, derived from *C. Wenyujin* (Er-Zhu or Yu-Jin) inhibits H22 tumor growth and enhances expression of histone H1 [19]. Ardipusilloside-I, a compound in *Ardisia pusilla* (Jiu-Jie-Long), inhibits tumor growth in H22 HCC [20]. Raddeanin A, a compound isolated from *Anemone raddeana* Regel (Liang-Tou-Jian), inhibits tumor growth in H22 HCC [21] (Figure 1, Table 2).

Indole-3-acetonitrile-4-methoxy-2-C-β-D-glucopyranoside, isolated from *Isatis indigotica* (Song-Lan) inhibits cell proliferation in HepG2 hepatoma cells [22]. Pinocembrin-7-*O*-[3-*O*-galloyl-4″,6″-hexahydroxydiphenoyl]-β-glucose (PGHG) and thonningianins A (Th A) from *Penthorum chinense* Pursh (Che-Gen-Cai) have been shown to have anti-HCC activities [23]. Annonaceous acetogenin-containing *Annona squamosa* (Li-Zhi-He) extract showed antiproliferative effects in liver cancer cells [24] (Table 2).

## 4. Induction of Apoptosis

Apoptosis is one of the most frequently studied cell processes for elucidating the effective mechanisms of herbal compounds against HCC. A triterpenoid and 20(*R*)-ginsenoside Rg3, isolated from *Panax ginseng*, induces apoptosis in liver cancer cells [25,26]. Gypenoside, a component of *Gynostemma pentaphyllum* (Jiao-Gu-Lan), induces apoptosis in hepatoma cells [27]. Isorhamnetin isolated from *Hippophae rhamnoides* (Sha-Ji) and liquiritigenin from *Glycyrrhiza uralensis* (Gan-Cao) induce apoptosis in HCC cells [28,29] (Table 2).

**Table 2:** Direct anticancer effects of herbal compounds against hepatocarcinoma.

Compounds	Herbs	Effects		Targets/Molecular Events		Ref.
Salvianolic acid B	*Salvia miltiorrhiza* Bunge (Dan-Shen)	↓ HepG2 cell proliferation		↓ CYP3A4 and CYP1A2, ↑ GST		[16]
Steroidal saponins	*Dioscorea bulbifera* (Huang-Du or Huang-Yao-Zi)	↓ SMMC7721 and Bel-7402 cell proliferation		Unknown		[17]
Davidiin	*Polygonum capitatum* (Tou-Hua-Liao)	↓ Hepatocellular tumor growth		↓ EZH2		[18]
β-Elemene	*Curcuma kwangsiensis* or *C. phaeocaulis* or *C. wenyujin* (E-Zhu)	↓ H22 tumor growth		↑ Histone H1		[19]
Ardipusilloside-I	*Ardisia pusilla* (Jiu-Jie-Long)	↓ SMMC-7721 tumor growth; ↓ invasion and metastasis in HCC		Unknown; ↓ MMP-9 and -2, ↑ Rac1 and E-cadherin		[20,30]
Raddeanin A	*Anemone raddeana* Regel (Liang-Tou-Jian)	↓ H22 tumor growth		Unknown		[21]
Indole-3-acetonitrile-4-methoxy-2-C-β- d-glucopyranoside	*Isatis indigotica* (Song-Lan)	↓ HepG2 cell proliferation		Unknown		[22]
Pinocembrin-7- *O*-[3-*O*-galloyl-4′′,6′′-hexahydroxydiphenoyl]-β-glucose and thonningianins A	*Penthorum chinense* Pursh (Che-Gen-Cai)	↓ Hepatocarcinoma cell growth		Unknown		[23]
20(*R*),22(xi),24(*S*)-dammar-25(26)-ene-3beta,6 alpha,12 beta,20,22,24-hexanol	*Panax ginseng* (Ren-Shen)	↓ proliferation, ↑ apoptosis, arrest cell cycle at the G1 phase		↑ p53 phosphorylation, activate caspase-3		[25]
20(*R*)-ginsenoside Rg3	*Panax ginseng* (Ren-Shen)	↑ apoptosis, ↓ liver cancer growth		↓ PCNA, ↑ TNF		[26]
Gypenoside	*Gynostemma pentaphyllum* (Jiao-Gu-Lan)	↓ proliferation, ↑ apoptosis in Hep3B and HA22T cells		Unknown		[27]
Isorhamnetin	*Hippophae rhamnoides* (Sha-Ji)	↓ proliferation, ↑ apoptosis in Bel-7402 cells		Unknown		[28]
Liquiritigenin	*Glycyrrhiza uralensis* (Gan-Cao)	↑ apoptosis, ↓ H22 tumor growth		Unknown		[29]
N-butylidenephthalide	*Angelica sinensis* (Dang-Gui)	↑ apoptosis in HepG2 and J5 cells, ↓ cell and tumor growth		↑ Nurr1, NOR-1, Nur77, CREB, caspase-9 and caspase-3, ↓ phosphor-AKT		[31]
*Lycium barbarum* polysaccharide	*Lycium barbarum* (Gou-Qi)	↓ proliferation, ↑ apoptosis, arrest cell cycle at S phase in QGY7703 cells		↑ Intracellular Ca2+		[32]
Apigenin	*Eclipta prostrate* (Mo-Han-Lian), *etc.*	↓ proliferation, ↑ apoptosis, arrest cell cycle at G2/M phase in Huh7 cells		↑ 1336 genes, ↓ 428 genes		[33]
Icariin	*Epimedium brevicornum* Maxim. (Yin-Yang-Huo)	↑ apoptosis in SMMC-7721 cells	↑ ROS, JNK, Bax/Bcl-2 and caspase			[34]
Icaritin	*Epimedium brevicornum* Maxim. (Yin-Yang-Huo)	↑ apoptosis in HepG2 cells	↑ JNK1, Bax/Bcl-2 and caspase-3			[35]
Oxymatrine	*Sophora flavescens* (Ku-Shen)	↓ proliferation, ↑ apoptosis, arrest cell cycle at S and G2/M phase in SMMC-7721 cells	↓ Bcl-2, ↑ p53			[36]
Scutellarin	*Scutellaria baicalensis* Georgi (Huang-Qin)	↓ proliferation, ↑ apoptosis in HepG2 cells	↓ ROS, STAT3, Bcl-XL and Mcl-1			[37]
Sarsasapogenin	*Anemarrhena asphodeloides* (Zhi-Mu)	↓ proliferation, ↑ apoptosis, arrest cell cycle at G2/M phase in HepG2 cells	Unknown			[38]
Pheophorbide a	*Scutellaria barbata* (Ban-Zhi-Lian)	↑ apoptosis in HepG2 and Hep3B cells	↓ Bcl-2, ↑ pro-caspase 3 and pro-caspase 9			[39]
Solamargine	*Solanum nigrum* (Long-Kui)	↓ proliferation, ↑ apoptosis, arrest cell cycle at G2/M phase in SMMC-7721 and HepG2 cells	↑ caspase-3			[40]
Ponicidin	*Rabdosia rubescens* (Dong-Ling-Cao)	↓ proliferation, ↑ apoptosis in QGY-7701 and HepG-2 cells	↓ Survivin and Bcl-2, ↑ Bax			[41]
Paeonol	*Paeonia suffruticosa* (Mu-Dan-Pi)	↓ tumor growth, ↑ apoptosis in HepA-hepatoma bearing mice	↓ Bcl-2, ↑ Bax, IL-2 and TNF-alpha			[42]
Cryptotanshinone, dihydrotanshinone, tanshinone I, tanshinone IIA	*Salvia miltiorrhiza* Bunge (Dan-shen)	↑ apoptosis in HepG2 cells	↑ ROS			[43]
Resveratrol-4-*O*-d-(2′-galloyl)-glucopyranoside	*Polygonum cuspidatum* (Hu-Zhang)	↓ proliferation, ↑ apoptosis in SMMC-7721 cells	↑ caspase-3 and -9, p-JNK, ↓ p-ERK			[44]
Tubeimoside I	*Bolbostemma paniculatum* (Tu-Bei-Mu)	↓ proliferation, ↑ apoptosis, arrest cell cycle at G2/M phase in HepG2 cells	↑ caspase-3 and -9, Bax/Bcl-2			[45]
Norcantharidin	Mylabris (Ban-Mao)	↓ proliferation, ↑ apoptosis in HepG2 cells	↑ ROS, caspase-3 and -9, and Bax, ↓ Bcl-2			[46]
Resveratrol-4-*O*-d-(2′-galloyl)-glucopyranoside	*Polygonum cuspidatum* (Hu-Zhang)	↓ proliferation, ↑ apoptosis in SMMC-7721 cells	↑ caspase-3 and -9, p-JNK, ↓ p-ERK			[44]
Toosendanin	*Melia toosendan* (Chuan-Lian-Zi)	↓ proliferation, ↑ apoptosis in SMMC-7721 and Hep3B cells	↑ Bax, ↓ Bcl-2			[47]
Honokiol	*Magnolia officinalis* (Hou-Po)	↑ apoptosis in liver cancer cells	↓ Bcl-X(L), Bcl-2, procaspase-3 and -9, ↑ MAPK and active caspase-3			[48]
Magnolol	*Magnolia officinalis* (Hou-Po)	↑ apoptosis in HepG2 cells	↑ caspase-3, -8, and -9, ↓ Bcl-2			[49]
Oleanolic acid and ursolic acid	The fruit of *Ligustrum lucidum* Ait. (Nü-zhen-zi), *Salvia chinensis* (Shi-Jian-Chuan), *Hedyotis diffusa* Willd. (Bai-Hua-She-She-Cao), *etc.*	↓ proliferation and adhesion, ↑ apoptosis in liver cancer cells		↑ caspase-3 and -8, ↓ Na(+)-K(+)-ATPase activity, VEGF and ICAM-1		[50]
Chrysophanol	*Rheum palmatum* L. or *R.tanguticum* Maxim.ex Balf. or *R.officinale* Baill. (Da-Huang)	↓ proliferation, ↑ necrosis in J5 cells		↓ ATP level, ↑ ROS and lactate dehydrogenase activity		[51]
Rhein	*Rheum palmatum* L. or *R.tanguticum* Maxim.ex Balf. or *R.officinale* Baill. (Da-Huang)	↑ apoptosis in HepG2 cells; ↓ proliferation, ↑ apoptosis, arrest cell cycle at S phase in Bel-7402 cells		↑ caspase-3; ↑ caspase-3, ↓ c-Myc		[52,53]
Vitexin compound 1	*Vitex negundo*(Huang-Jing)	↓ proliferation, ↑ apoptosis in liver cancer cells		↑ caspase-3, -8 and -9, FOXO3a, Bim, TRAIL, DR4 and DR5, ↓ phosphorylation of AKT and ERK1/2		[54]
Quercetin	*Bupleurum chinense* (Chai-Hu), *Euphorbia lunulata* Bunge (Mao-Yan-Cao) and *Taxillus chinensis* (Sang-Ji-Sheng), *etc.*	↓ proliferation, ↑ apoptosis in HA22T/VGH cells		↑ ROS		[55]
Gambogic acid	*Garcinia hanburyi* (Teng-Huang)	↓ proliferation, ↑ apoptosis in SMMC-7721 cells		↑ Bax, ↓ Bcl-2		[56]
Flavonoids	*Polygoni Orientalis* Fructus (Shui-Hong-Hua-Zi )	↓ proliferation, ↑ apoptosis, arrest cell cycle at S phase in SMMC-7721 cells		Unknown		[57]
TSP02	*Ardisia japonica* (Zi-Jin-Niu)	↓ proliferation, migration and invasiveness, ↑ apoptosis in HepG2 cells		↓ CDK1, 2, 4, and TGF-beta1, ↑ Caspase-8 and E-cadherin		[58]
Bufothionine	*Bufonis Venenum* (Chan-Su)	↓ proliferation, ↑ arrest cell cycle at G2/M phase in hepatocarcinoma cells		Unknown		[59]
Oridonin	*Rabdosia rubescens* (Dong-Ling-Cao)	↓ proliferation, ↑ apoptosis, arrest cell cycle at G2/M phase in HepG2 cells		↑ p-JNK, p-p38, p-p53, p21, cyclin B1/p-Cdc2 (Tyr15), caspase-9 and -3, ↓ p-ERK		[60]
Curcumol	*Curcuma kwangsiensis* or *C. phaeocaulis* or *C. wenyujin* (E-Zhu)	↓ proliferation, arrest cell cycle at G1 phase in HepG2 cells		↑ pRB1, cyclin D1, CDK2, CDK8, p27KIP1, p53 and p21WAF1, ↓ cyclin A1		[61]
Saikosaponin d	*Bupleurum chinense* (Chai-Hu)	↓ proliferation, ↑ apoptosis, arrest cell cycle at G1 phase in HepG2 and Hep 3B cells		↑ p53, p21/WAF1, Fas/APO-1, mFasL, sFasL, Bax and IkappaBalpha, ↓ NF-kappaB and Bcl-XL		[62]
Waltonitone	*Gentiana waltonii* (Chang-Geng-Qin-Jiao)	↓ proliferation, arrest cell cycle at S phase in Bel-7402 cells		↑ Akt and ERK1/2 phosporylation		[63]
Nobiletin	*Citrus aurantium* (Zhi-Shi)	↓ proliferation, ↑ apoptosis, arrest cell cycle at G2 phase in SMMC-7721 cells		↑ Bax and caspase-3, ↓ Bcl-2 and COX-2		[64]
Matrine	*Sophora flavescens* (Ku-Shen)	↓ proliferation, ↑ apoptosis and autophagy, arrest cell cycle at G1 phase in HCC cells; ↓ invasion in SMMC-7721 cells			↑ Bax/Bcl-2 and Beclin 1; ↓ MMP-9 and NF-κB	[65,66,67]
Berberine	*Coptis chinensis* Franch. (Huang-Lian) or *Phellodendron chinense* Schnied. (Huang-Bai)	↑ apoptosis, arrest cell cycle at G1 phase in HuH7 cells; ↑ apoptosis and autophagy in HepG2 cells			↓ PCNA, Bid and Bcl-2, ↑ caspase-3 and -7; ↑ AMPK, ↓ mTORC1	[68,69]
Baicalein	*Scutellaria baicalensis* Georgi (Huang-Qin)	↑ apoptosis, arrest cell cycle at G2/M phase in J5 cells; ↓ proliferation, ↑ apoptosis and autophagy in SMMC7721 cells			↑ caspase-9 and -3, and Bax/Bcl-2 ratio; ↑ Βeclin 1, ↓ CD147	[70,71]
Oroxylin-A	*Scutellaria baicalensis* Georgi (Huang-Qin)	↓ proliferation, ↑ apoptosis and autophagy in HepG2 cells; reverse drug resistance and enhance apoptosis inducing effect of Paclitaxel in drug resistant HepG2 cells			Induction of Bax translocation, activation and oligomerization, ↑ Βeclin 1, ↓ PI3K-PTEN-Akt-mTOR signaling pathway; ↓ Integrinβ1	[72,73,74]
Shikonin	*Lithospermum erythrorhizon* (Zi-Cao)	↑ apoptosis in Huh7 and BEL7402 cells; ↑ autophagy in HCC cells; ↓ proliferation and migratory ability on HepJ5 and Mahlavu cells			↑ ROS, ↓ Akt and RIP1/NF-κB; ↑ ROS and ERK, ↓ RIP pathway; ↓ MMP-2 and -9, vimnetin, AKT and IκB phosphorylation, NF-κB	[75,76,77]
Curcumin	*Curcuma kwangsiensis* (Yu-Jin or Er-Zhu), *C. phaeocaulis* (Yu-Jin or Er-Zhu), *C. wenyujin* (Yu-Jin or Er-Zhu), *C. longa* (Yu-Jin or Jiang-Huang), *etc.*	↑ apoptosis in Huh7 cells; ↑ apoptosis and autophagy in HepG2 cells; ↓ proliferation, EMT and migration in hypoxic HepG2 cells			↑ p38, FasL and caspase-3; ↑ caspase-3, ↓ Bcl-2/Bax; ↓ HIF-1alpha	[78,79,80]
Resveratrol	*Polygonum cuspidatum* (Hu-Zhang), *etc.*	↓ proliferation, ↑ apoptosis in Hepa 1-6 cells; ↓ proliferation, ↑ apoptosis and autophagy, arrest cell cycle at S phase in HuH7 cells; ↓ invasion in HCC cells			↑ROS and caspase-3; ↑ p21/WAF1, Atg5, Atg7, Atg9, and Atg12, ↓ cyclin E, cyclin A, CDK2, phospho-ERK and phospho-p38; ↓ MMP-9	[81,82,83,84]
Bufalin and cinobufagin	Toad skin and venom	↑ apoptosis in HepG2 cells (Bufalin and cinobufagin); ↑ apoptosis and autophagy in HepG2 cells (Bufalin)			↑ Fas, Bax and Bid, caspase-3, -8, -9 and -10, ↓ Bcl-2 (Bufalin and cinobufagin; ↑ Βeclin 1 and AMPK phosphorylation, ↓ p62 and mTOR signaling (Bufalin)	[85,86]
Tetrandrine	*Stephania tetrandra* (Han-Fang-Ji)	↓ proliferation, ↑ apoptosis and autophagy, arrest cell cycle at G2/m phase in liver cancer cells			↑ ROS, ERK MAP kinase and ATG7, ↓ Akt	[87,88,89]
Arenobufagin	Toad venom	↑ apoptosis and autophagy in HepG2 cells			↑ Bax/Bcl-2, ↓ PI3K/Akt/mTOR pathway	[90]
Allicin	Garlic	↓ proliferation, ↑ autophagy in HepG2 cells			↑ AMPK/TSC2 and Beclin-1 signaling, ↓ p53, the PI3K/mTOR signaling and Bcl-2	[91]
Galangin	*Alpinia officinarum* Hance (Gao-Liang-Jiang)	↓ proliferation, ↑ apoptosis and autophagy in HepG2 cells			↑ p53	[92]
Kaempferol	*Euphorbia lunulata* Bunge. (Mao-Yan-Cao)	↓ proliferation, ↑ autophagy, arrest cell cycle at G2/M phase in SK-HEP-1 cells			↑ p-AMPK, LC3-II, Atg 5, Atg 7, Atg 12 and beclin 1, ↓CDK1, cyclin B, p-AKT and p-mTOR	[93]
EGCG	Tea	Inhibit autophagy to enhance anti-cancer effects of doxorubicin in Hep3B cells			↓ Βeclin 1 and Atg5	[94]
Elemene injection	*Curcuma kwangsiensis* or *C. phaeocaulis* or *C. wenyujin* (E-Zhu)	Induce autophagy and prevent HepG2 cells from undergoing apoptosis			↓ Bcl-2/Bax and LC3 I/LC3 II ratio	[95]
Ganoderiol F	*Ganoderma* *amboinense* (Lu-Jiao-Ling-Zhi)	↓ proliferation, ↑ cell senescence in HepG2 cells			↑ EKR and p16	[96]
Arecoline	*Areca catechu* L. (Bing-Lang)	↑ anoikis in HA22T/VGH cells			↑ Bax, caspase-3 and Rho/Rock activation, ↓ beta1-integrin, IL-6, STAT3 and p190RhoGAP phosphorylation, SHP2, Bcl-XL and Bcl-2	[97]
Tanshinone II-A	*Salvia miltiorrhiza* Bunge (Dan-shen)	↓ proliferation, ↑ apoptosis, arrest cell cycle at G0/G1 phase in SMMC-7721 cells; ↓ EMT and metastasis in HCC; ↓ migration and invasion in HCC cells			↓ Bcl-2 and c-myc, ↑ Fas, Bax and p53; ↑ VEGFR1/PDGFR; ↓ MMP-2 and -9, NF-κB	[98,99,100]
Dihydroartemisinin	*Artemisia annua* L. (Qing-Hao)	↓ proliferation, ↑ apoptosis, arrest cell cycle at G2/M phase in HCC cells; ↓ invasion and metastasis in HCC cells			↑ p21, caspase-9 and -3 , Noxa and active Bak, ↓ cyclin B, CDC25C and Mcl-1; ↓ MMP2, ↑ TIMP2, Cdc42 and E-cadherin	[101,102]
Cordycepin	*Cordyceps sinensis* (Dong-Chong-Xia-Cao)	↓ proliferation, EMT and migration/invasion			↓ integrin α3, integrin α6, integrin β1 and phosphorylated FAK	[103]
Polysaccharides	Huaier	↓ proliferation, EMT, adhesion, migration and invasion in MHCC97-H cells			↓ AEG-1	[104]
Platycodin D	*Platycodon grandiflorum* (Jie-Geng)	↓ proliferation, adhesion, migration and invasion in HCC cells			↑ Bax, ↓ survivin	[105]
Isofraxidin	*Acanthopanax senticosus* (Ci-Wu-Jia)	↓ invasion in HCC cells			↓ MMP-7 and ERK1/2	[106]
β-Ionone	*Aucklandia lappa* Decne or *Vladimiria souliei* (Franch.) Ling (Mu-Xiang)	↓ invasion, migration and adhesion in SK-Hep-1 cells			↓ MMP-2 and -9, urokinase-type plasminogen activator activities, FAK, Rho, Rac1 and Cdc42, ↑ TIMP-1 and -2, plasminogen activator inhibitor-1 and nm23-H1	[107]
Hesperidin	*Citrus reticulata* Blanco (Chen-Pi)	↓ acetaldehyde-induced cell invasion in HepG2 cells			↓ MMP-9, NF-kappaB, AP-1, JNK, and p38 signaling pathways	[108]
Astragalosides, *astragalus* polysaccharide and salvianolic acids	*Astragalus membranaceous* (Huang-Qi), *Salvia miltiorrhiza* Bunge (Dan-shen)	↓ TGF-beta(1)-induced cell invasion in HepG2 cells			Modulating TGF-beta/Smad signaling	[109]
*Astragalus* polysaccharides	*Astragalus membranaceous* (Huang-Qi)	↑anti-tumor effect of Adriamycin in H22 hepatocarcinoma			↑ IL-1α, IL-2, IL-6 and TNF-α, ↓ IL-10 and MDR1	[110]
Tetramethylpyrazine	*Ligusticum chuanxiong* Hort (Chuan-Xiong)	Reverse multidrug resistance in BEL-7402/ADM cells			↓ MDR1, MRP2, MRP3 and MRP5	[111]
Epicatechin gallate and epigallocatechin gallate	Tea	Increase intracellular DOX accumulation and enhance DOX-induced cell killing activities against BEL-7404/DOX cells			↓ MDR1	[112]
Hedyotiscone A	*Hedyotis corymbosa* (San-Fang-Hua-Er-Cao)	↑ apoptosis in multidrug-resistant hepatocellular carcinoma cells			↑ caspases-3, -7 and -9	[113]
Polyphyllin D	*Paris polyphylla* Sm. (Chong-Lou)	↑ apoptosis in multi-drug resistant HepG2 cells			Mitochondrial dysfunction	[114]
Ursolic acid	*Gardenia jasminoides* Ellis (Zhi-Zi ), *Prunella vulgaris* (Xia-Ku-Cao), *Hedyotis diffusa* Willd. (Bai-Hua-She-She-Cao), *etc.*	↑ apoptosis in doxorubicin-resistant human hepatoma cells			↑ Bak and apoptosis-inducing factor	[115]
Pseudolaric acid B	*Pseudolarix kaempferi* (Tu-Jin-Pi)	↑ apoptosis and arrest cell cycle at G2/M phase in conventional and P-gp-overexpressing hepatocarcinoma cells			Disrupts cellular microtubule networks and inhibits the formation of mitotic spindles	[116]
Imperatorin	*Angelica dahurica* (Bai-Zhi)	↑ apoptosis in multidrug-resistant liver cancer cells			↑ proteosome-dependent Mcl-1 degradation to release Bak and Bax	[117]

↓ Inhibit or down-regulate, ↑ promote or up-regulate.

N-Butylidenephthalide, derived from *Angelica sinensis* (Dang-Gui), is effective in inducing apoptosis in HCC cells [31]. Polysaccharide from *Lycium barbarum* (Gou-Qi) induces apoptosis in liver cancer cells [32]. Apigenin, a common compound abundantly present in *Eclipta prostrate* (Mo-Han-Lian) and other herbs, fruits or vegetables, induces apoptosis and arrests cell cycle at G2/M phase in HCC cells [33]. Icariin and icaritin, isolated from *Epimedium brevicornum* Maxim. (Yin-Yang-Huo), up-regulate JNK and Bax/Bcl-2 to induce apoptosis in liver cancer cells [34,35] (Table 2).

Matrine and oxymatrine from *Sophora flavescens* (Ku-Shen) induce apoptosis in HCC cells [36,65] (Figure 1). Berberine, a compound from *C. chinensis* Franch. (Huang-Lian), induces apoptosis in liver cancer cells via the mitochondrial pathway [68]. Scutellarin, baicalein, and oroxylin-A, derived from *Scutellaria baicalensis* Georgi (Huang-Qin), induce apoptosis in HCC cells [37,70,72]. Sarsasapogenin from *Anemarrhena asphodeloides* (Zhi-Mu) induces apoptosis and G2/M cell cycle arrest in liver cancer cells [38] (Table 2).

Pheophorbide A, a component purified from *S. barbata* (Ban-Zhi-Lian), inhibits Bcl-2 expression and induces apoptosis via a mitochondria-mediated intrinsic pathway in HCC cells [39]. Solamargine purified from *Solanum nigrum* (Long-Kui) induces apoptosis and G2/M cell cycle arrest in SMMC-7721 cells [40] (Figure 1). Ponicidin, a compound from *Rabdosia rubescens* (Dong-Ling-Cao), induces apoptosis accompanied by down-regulation of Survivin and Bcl-2 and up-regulation of Bax in HCC cells [41] (Figure 1). Shikonin from *Lithospermum erythrorhizon* (Zi-Cao) induces apoptosis in HCC cells through generation of reactive oxygen species (ROS) and down-regulation of Akt and RIP1/NF-κB pathways [75]. Paeonol from *Paeonia suffruticosa* (Mu-Dan-Pi) induces apoptosis in HCC [42] (Table 2).

Curcumin, a component in *Curcuma kwangsiensis* (Yu-Jin or Er-Zhu) or *C. phaeocaulis* (Yu-Jin or Er-Zhu), *C. wenyujin* (Yu-Jin or Er-Zhu), *C. longa* (Yu-Jin or Jiang-Huang), induces apoptosis via p38 activation [78]. Cryptotanshinone, dihydrotanshinone, tanshinone I and tanshinone IIA derived from *S. miltiorrhiza* Bunge (Dan-Shen), and resveratrol and resveratrol-4-*O*-D-(2′-galloyl)-glucopyranoside from Hu-Zhang (*Polygunum cuspidatum*), induce apoptosis in HCC cells [43,44,81,98]. Tubeimoside I, an ingredient derived from *Bolbostemma paniculatum* (Tu-Bei-Mu), up-regulates Bax/Bcl-2 and induces intrinsic apoptosis in hepatoma cells [45] (Table 2).

Norcantharidin potently increases ROS production, down-regulates Bcl-2, up-regulates Bax, and activates caspase-3 and -9 to induce apoptosis in liver cancer cells [46]. Bufalin and cinobufagin, isolated from toad skin and venom, induce apoptosis via Fas- and mitochondria-mediated pathways in HCC cells [85]. Tetrandrine, a compound isolated from *Stephania tetrandra* (Han-Fang-Ji), induces apoptosis in HCC cells by activating ROS production and repressing Akt activity [87]. Toosendanin, isolated from *Melia toosendan* (Chuan-Lian-Zi), induces mitochondria-dependent apoptosis in HCC cells [47]. Honokiol and magnolol, isolated from *Magnolia officinalis* (Hou-Po) induce apoptosis in liver cancer cells [48,49] (Table 2).

Oleanolic acid and ursolic acid, compounds widely present in the fruit of *Ligustrum lucidum* Ait. (Nü-zhen-zi), *Salvia chinensis* (Shi-Jian-Chuan), *H. diffusa* Willd. (Bai-Hua-She-She-Cao) and other herbs, have demonstrated apoptosis-inducing effects in HCC cells [50]. Chrysophanol and rhein, compounds isolated from *Rheum palmatum* L. or *R. tanguticum* Maxim.ex Balf. or *R. officinale* Baill. (Da-Huang), induce necrosis or apoptosis in HCC cells [51,52]. Vitexin compound 1 from the seed of *Vitex negundo* (Huang-Jing) induces apoptosis in HCC cells via activation of FOXO3a and down-regulation of AKT and ERK1/2 phosphorylation [54] (Table 2).

Quercetin, a common compound in many herbs such as *B. chinense* (Chai-Hu), *Euphorbia lunulata* Bunge (Mao-Yan-Cao) and *Taxillus chinensis* (Sang-Ji-Sheng), induces apoptosis in HCC cells and is related to ROS generation [55]. Gambogic acid, a compound in *Garcinia hanburyi* (Teng-Huang), induces apoptosis in HCC with relatively less adverse effects on normal hepatocytes [56]. Flavonoids isolated from *Polygoni Orientalis* Fructus (Shui-Hong-Hua-Zi) induce apoptosis and G2/M phase cell cycle arrest in HCC cells [57]. TSP02, a triterpenoid saponin from *Ardisia japonica* (Zi-Jin-Niu) has demonstrated apoptosis-inducing effects in hepatoma cells [58] (Table 2).

## 5. Cell Cycle Arrest

Cancer is characterized by uncontrolled proliferation cycle and stopping the cell cycle is an ideal approach for cancer treatment. Some herbs are effective at arresting the cell cycle. Bufothionine inhibits cell proliferation and arrests cell cycle at G2/M phase in liver cancer cells [59]. Rhein, a component of *R. palmatum* L, *R. tanguticum* Maxim.ex Balf. or *R. officinale* Baill. (Da-Huang), is effective in inhibiting cell growth, inducing apoptosis and arresting cell cycle at S phase in HCC cells [53]. Oridonin, a compound from *R. rubescens* (Dong-Ling-Cao), induces G2/M cell cycle arrest and apoptosis in HepG2 cells through activation of the MAPK and p53 pathways [60] (Figure 1, Table 2).

Curcumol, a compound present in *C. phaeocaulis*, *C. wenyujin* or *C. kwangsiensis* (Er-Zhu), inhibits cell proliferation and induces G0 phase cell cycle arrest by activating the p53/RB pathway that involves cyclin A1, CDK2, CDK8, p21WAF1 and p27KIP1 in HCC cells [61] (Figure 1). Tanshinone IIA, isolated from *S. miltiorrhiza* Bunge (Dan-Shen), inhibits proliferation and induces G2/M cell cycle arrest accompanied by up-regulation of calreticulin, caspase-12 and GADD15, and down-regulation of Bcl-2, Cdc25c and Cdc2 in HCC cells [118]. Resveratrol, a compound can be found in *P. cuspidatum* (Hu-Zhang), induces cell cycle arrest, apoptotic and autophagic cell death in liver cancer cells [82] (Table 2).

Tetrandrine, a compound isolated from *S. tetrandra* (Han-Fang-Ji), inhibits proliferation and arrests cell cycle at G2/M phase in HCC cells [88]. Dihydroartemisinin, a semi-synthetic derivative of artemisinin isolated from *Artemisia annua* L. (Qing-Hao), induces apoptosis, and G2/M cell cycle arrest through induction of p21 and inhibition of cyclin B and CDC25C in liver cancer cells [101]. Saikosaponin d, a compound isolated from *B. chinense* (Chai-Hu), has demonstrated potency in inducing apoptosis and G0 cell cycle arrest by up-regulating p53 and p21/WAF1 in HCC cells [62] (Table 2).

Waltonitone, a compound isolated from *Gentiana waltonii* (Chang-Geng-Qin-Jiao), is effective in inhibiting cell proliferation and inducing S phase cell cycle arrest through activation of Akt and ERK1/2 phosporylation in liver cancer cells [63]. Nobiletin, a compound from *Citrus aurantium* (Zhi-Shi) or other herbs, induces apoptosis and G2 phase cell cycle arrest in HCC cells [64]. *S. nigrum* L. (Long-Kui) polyphenolic extract induces apoptosis and G2/M cell cycle arrest by down-regulating CDC25A, CDC25B and CDC25C in hepatoma cells [119]. Triterpene-enriched extracts from *Ganoderma lucidum* (Ling-Zhi) could suppress PKC, activate JNK and p38 MAPK to prolong G2 phase and inhibit cell growth in Huh-7 cells [120] (Table 2).

## 6. Induction of Autophagy

Autophagy, type II programmed cell death, is a process in which organelles and proteins are sequestered and subsequently degraded through fusion with lysosomes, and has been recognized as a target for HCC treatment [121,122]. Tetrandrine, isolated from *S. tetrandra* (Han-Fang-Ji), promotes ROS generation and activates ERK MAP kinase to induce autophagy in HCC cells [89] (Figure 1). Shikonin, a naphthoquinone from *L. erythrorhizon* (Zi-Cao), induces autophagy in HCC cells via ROS production and ERK activation [76]. Matrine, a compound from *S. flavescens* Ait. (Ku-Shen), induces apoptosis and autophagy in HepG2 cells [66] (Table 2).

Baicalein, a compound from *S. baicalensis* Georgi (Huang-Qin), down-regulates CD147 and induces autophagy in liver cancer cells [71] (Figure 1). Oroxylin A, another compound from *S. baicalensis* Georgi (Huang-Qin), induces autophagy by suppressing PI3K-PTEN-Akt-mTOR signaling in HepG2 cells [73]. Berberine, a compound isolated from *C. chinensis* Franch. (Huang-Lian) and other herbs, is effective at inducing apoptosis and autophagy associated with AMPK activation and mTORC1 inhibition [69] (Table 2).

Bufalin, a component from toad skin, has demonstrated efficacy in the induction of AMPK-dependent autophagy accompanied by enhanced Beclin-1 expression, and decreased p62 expression and mTOR signaling in HepG2 cells [86] (Figure 1). Arenobufagin, a natural bufadienolide from toad venom, is a potent inducer of apoptosis and autophagy by down-regulating PI3K/Akt/mTOR pathway in HepG2/ADM hepatoma cells [90]. Curcumin, a common compound in herbs belonging to the plant genus *Curcuma*, when combined with Adriamycin, induces apoptosis and autophagy in Hep G2 cells by down-regulating Bcl-2/Bax [79] (Table 2).

Allicin, a major phytochemical found in garlic, is effective at inducing autophagy in HepG2 cells by decreasing the level of cytoplasmic p53, PI3K/mTOR signaling and Bcl-2, and up-regulating the expression of AMPK/TSC2 and Beclin-1 [91]. Galangin, a component of *Alpinia officinarum* Hance (Gao-Liang-Jiang), has demonstrated potency in up-regulating p53 and inducing apoptosis and autophagy in HepG2 cells [92]. Kaempferol, a common compound in *E. lunulata* Bunge. (Mao-Yan-Cao) and other herbs, induces G2/M cell cycle arrest, apoptosis, and autophagy, and related to CDK1/cyclin B expression and AMPK and AKT signaling pathways [93] (Table 2).

However, in a different context, autophagy may promote cell death and contribute to anticancer therapeutic response, or inhibit cell death and thus contribute to drug resistance [123]. Inhibition of autophagy may enhance chemotherapy or target therapy-induced cell death in HCC cells [124,125]. As an autophagy inhibitor, epigallocatechin-3-*O*-gallate (EGCG) strengthens doxorubicin (DOX)-mediated anticancer effects in hepatoma cells [94]. As an autophagy inducer, elemene injection (a herbal injection) protects hepatoma cells from apoptosis induced by apatinib or serum-free starvation [95]. These observations suggest that autophagy-inducing herbal compounds need to be more thoroughly investigated in combination with other treatments such as chemotherapy and targeted therapy Table 2.

## 7. Induction of Cell Senescence

Cell senescence is a stable, irreversible cell cycle arrest triggered by a variety of stimuli. Induction of senescence has been suggested as novel approach for HCC treatment [126]. Ganoderiol F, a tetracyclic triterpene from *Ganoderma amboinense*, activates ERK and up-regulates p16 to induce cell senescence in hepatoma HepG2 cells [96] (Figure 1, Table 2).

*L. lucidum* Ait. Fruit (Nü-zhen-zi) up-regulates p21, activates caspases-8, -9 and -3 to induce apoptosis, and down-regulates RB phosphorylation to induce cell senescence in heaptocarcinoma cells [127]. The known compounds in *L. lucidum* Ait. fruit include oleanolic acid, ursolic acid and tartaric acid*.* The compound(s) responsible for inducing cell senescence need further study.

Liver Yin tonifying formula (LYTF) activates caspases-8, -9 and -3 to induce apoptosis, and up-regulates p16 and p21 and down-regulates RB phosphorylation to induce cell senescence in Bel-7402 cells [128]. The known compounds in LYTF include oleanolic acid, ursolic acid and emodin. The compound(s) responsible for inducing cell senescence need further investigation.

## 8. Induction of Anoikis

Anoikis, an apoptotic process occurring when a cell detaches from extracellular matrix, is associated with cell survival in suspension and metastasis of HCC [129]. Arecoline, an alkaloid from *Areca catechu* L. (Bing-Lang), induces anoikis in HA22T/VGH cells by inhibiting STAT3 and increasing RhoA/Rock activation [97] (Figure 1, Table 2).

*P. cuspidatum* (Hu-Zhang), a common herb used for HCC treatment, activates caspase-3 and -9 and induces anoikis in human HCC cells accompanied by ROS generation and FAK down-regulation [130]. The known compounds in Hu-Zhang include resveratrol, emodin, polydatin and physcion. However, the compound(s) contributing to Hu-Zhang-induced anoikis need further study.

Modified Yi Guan Jian (MYGJ) may activate caspase-3, -8 and -9, inhibit the expression and phosphorylation of p38 MAPK, and induce anoikis in human HCC cells [131]. The known compounds in MYGJ include coumarin, glucosides, betane and toosendanin. Further study is needed to identify the compound(s) responsible for MYGJ-induced anoikis.

## 9. Inhibition of Epithelial-Mesenchymal Transition (EMT)

EMT is a biological process in which polarized epithelial cells (including epithelial cancer cells) switch to a fibroblastoid or mesenchymal cellular phenotype [132]. During EMT, the epithelial marker E-cadherin (E-cad) is down-regulated and mesenchymal markers, such as N-cadherin (N-Cad) and vimentin, are up-regulated. EMT contributes to cancer metastasis by enhancing cell migration and invasiveness. EMT also contributes to drug resistance [133].

Tanshinone IIA, a compound isolated from *S. miltiorrhiza* Bunge (Dan-Shen), has demonstrated effects in inhibiting EMT and metastasis in HCC after palliative resection [99] (Figure 1). Curcumin, a common herbal compound in herbs of the plant genus *Curcuma*, inhibits hypoxia-induced EMT in hepatoma HepG2 cells [80]. Cordycepin, a compound isolated from *Cordyceps sinensis* (Dong-Chong-Xia-Cao), may up-regulate E-cad and down-regulate integrins and phosphorylation of FAK to inhibit proliferation, migration and invasion in HCC cells [103] (Figure 1 Table 2).

Huaier polysaccharides are effective in inhibiting proliferation, adhesion, migration and invasion in HCC cells accompanied by EMT inactivation as indicated by marker gene expression [104] (Figure 1, Table 2). Songyou Yin, a formula composed of five herbs, has reported efficacy in attenuating EMT and inhibiting metastatic potential in residual HCC resulted from oxaliplatin treatment [134]. The known compounds in Songyou Yin include tanshinone IIA and astragaloside IV.

## 10. Inhibition of Metastasis

Chinese herbal compounds have demonstrated potency in inhibiting metastatic potential in HCC cells, such as adhesion, migration, invasion, and metastasis. Matrine, a component of Ku-Shen (*S. flavescens* Ait.), down-regulates MMP-9 and NF-κB and inhibits invasion in liver cancer cells [67]. Shikonin, an ingredient of *L. erythrorhizon* (Zi-Cao), inhibits migration in HCC cells via down-regulation of vimentin, MMP-2 and MMP-9 [77]. Artemisinin, from *A. annua* L. (Qing-Hao), inhibits HCC metastasis by down-regulation of MMP2 and phosphorylation of p38 and ERK1/2, and up-regulation of TIMP2, Cdc42 and E-cadherin [102] (Figure 1, Table 2).

Gekko sulfated polysaccharide-protein complex inhibits HCC cell migration through calcium-mediated regulation of actin cytoskeleton reorganization [135]. Tanshinone IIA, a component isolated from *S. miltiorrhiza* (Dan-Shen), inhibits invasion and metastasis of HCC cells by inhibiting MMP-2 and MMP-9 activities and blocking NF-κB activation [100]. Resveratrol inhibits adhesion, migration, invasion and MMP-9 expression in liver cancer cells [83,84] (Figure 1). Platycodin D, a compound isolated from *Platycodon grandiflorum* (Jie-Geng), inhibits cell adhesion, migration and invasion of HCC cells [105]. Ardipusilloside I, a compound isolated from *A. pusilla* (Jiu-Jie-Long), inhibits liver cancer survival, invasion and metastasis by down-regulating MMP-9 and MMP-2 and activating Rac 1 to enhance E-cadherin activity [30] (Table 2).

Isofraxidin, isolated from *Acanthopanax senticosus* (Ci-Wu-Jia), inhibits MMP-7 expression and cell invasion in human hepatoma cells [106] (Figure 1). β-Ionone, a compound found in *Aucklandia lappa* Decne, *Vladimiria souliei* (Franch.) Ling (Mu-Xiang) and other herbs, inhibits cell invasion, migration and adhesion in HCC cells [107]. Hesperidin, a compound can be found in *Citrus reticulata* Blanco (Chen-Pi) and other herbs, has reported efficacy in inhibiting acetaldehyde-induced MMP-9 expression and cell invasion in HCC cells [108] (Table 2).

In addition to single compound, multiple herbal ingredients can be combined and used as a formula. Compound *Astragalus* and *Salvia miltiorrhiza* extract (CASE), a herbal component formula comprising astragalosides, astragalus polysaccharide and salvianolic acids, inhibits TGF-β1 mediated invasion in hepatoma HepG2 cells by modulating TGF-β/Smad signaling [109] (Table 2).

## 11. Targeting Drug Resistance

Drug resistance contributes to chemotherapy-refractory HCC [136]. The search for effective herbal components to reverse drug resistance has become a research focus area in liver cancer studies. *Astragalus membranaceus* (Huang-Qi) polysaccharides enhance the anti-tumor effects of adriamycin in H22 hepatocarcinoma by up-regulating IL-1α, IL-2, IL-6 and TNF-α, as well as down-regulating IL-10 and MDR1 [110]. Astragaloside II, another component from *A. membranaceus* (Huang-Qi), is effective in enhancing cytotoxicity of 5-fluorouracil (5-Fu) in 5-Fu-resistant HCC cells accompanied by down-regulation of P-gp, phosphorylation of ERK1/2, p38 and JNK [137] (Figure 1, Table 2).

Oroxylin A, a compound isolated from *S. baicalensis* Georgi (Huang-Qin) potently inhibits integrin β1, reverses drug resistance and enhances apoptosis-inducing effect of paclitaxel in drug-resistant HepG2 cells [74] (Figure 1). Tetramethylpyrazine, a bioactive constituent isolated from *Ligusticum chuanxiong* Hort (Chuan-Xiong), down-regulates MDR1, MRP2, MRP3 and MRP5 in adriamycin-resistant HepG2 cells [111] (Figure 1). Epicatechin gallate (ECG) and epigallocatechin gallate (EGCG) inhibits MDR1 expression, increases intracellular DOX accumulation and enhance DOX-induced cytotoxicity in BEL-7404/DOX cells [112] (Table 2).

In addition to reversing drug resistance, some herbal compounds have direct effects against drug-resistant liver cancer cells. Hedyotiscone A, a compound isolated from *H. corymbosa* (San-Fang-Hua-Er-Cao), activates caspases-3, -7 and -9 to induce apoptosis in multidrug resistant HCC cells [113]. Polyphyllin D, derived from *Paris polyphylla* Sm. (Chong-Lou), induces apoptosis in multidrug resistant HepG2 cells via mitochondrial dysfunction [114]. Ursolic acid, a common component in multiple herbs, activates Bak and promotes release of apoptosis-inducing factor to induce apoptosis in doxorubicin-resistant human hepatoma cells [115] (Table 2).

Pseudolaric acid B, a compound present in *Pseudolarix kaempferi* (Tu-Jin-Pi), may disrupt cellular microtubule networks and inhibit mitotic spindle formation to induce apoptosis and G2/M cell cycle arrest in conventional and P-gp-overexpressing HCC cells [116]. Imperatorin, a compound isolated from *Angelica dahurica* (Bai-Zhi), induces proteosome-dependent Mcl-1 degradation to release Bak and Bax and triggers apoptosis in multidrug-resistant liver cancer cells [117] (Table 2).

## 12. Regulation of Immune Function

The major cell populations for cellular immunity against cancer include CD4+ T helper (Th) and CD8+ T lymphocytes. CD4+ Th1 cells produce cytokines, such as IL-2 and IFN-γ, to evoke cell-mediated immunity or phagocyte-dependent inflammation. CD4+ Th2 cells secrete cytokines, such as IL-4 and IL-6, and are correlated with humoral immunity. Antigen-presenting cells, such as dendritic cells (DCs) and macrophages, processed antigens are necessary to prime CD4+ and CD8+ T cells to elicit antigen-specific immune response.

*L. barbarum* (Gou-Qi) polysaccharides increase CD4+ and CD8+ T cells in H22 hepatoma bearing mice [138]. Polysaccharides isolated from *A. annua* L. (Huang-Hua-Hao) increase CD4+ and CD8+ T cells and IFN-γ and IL-4 secretion in HCC bearing mice, and induce cancer cell apoptosis in HCC [139] (Figure 1, Table 3).

Gastrodin, isolated from *Gastrodia elata* Blume (Tian-Ma), up-regulates NF-κB, IL-2 and Bcl-2 in CD4+ T cells, and enhances cytotoxic activities of natural killer (NK) and CD8+ T cells against H22 hepatic cancer cells [140]. Shikonin, a major component of *L. erythrorhizon* (Zi-Cao) and *Arnebia euchroma* (Ruan-Zi-Cao), is effective in increasing CD3+ and CD19+ lymphocytes, and improving NK activities, lymphocyte transformation and IL-2 production in hepatoma HepA22 bearing mice [141] (Figure 1, Table 3).

The proteins extracted from Lei-Wan (*Omphalia lapidesces*) are effective in increasing spleen mass and IFN-γ production in H22 HCC bearing mice [142] (Table 3). *Eupolyphaga sinensis* Walker (Tu-Bie-Chong) ethanol extract enhances Th1 type cytokines (TNF-α and IFN-γ) production, and induces cell apoptosis in H22 HCC bearing mice [143].

DCs are important antigen-presenting cells for anticancer immunity. *L. barbarum* (Gou-Qi) polysaccharides promote DCs to stimulate allogeneic lymphocyte proliferation, produce IL-12p70 and IFN-γ and may relate to NF-κB expression [144]. Chen *et al.* have found that HCC SMMC-7721 cells may impair the biorheological properties of DCs, such as cell deformability, migration, and electrophoresis mobility, and altered organizations of cytoskeletal proteins. *Gekko Chinensis* (Tian-Long) sulfated polysaccharide-protein complex partially restores the defective biorheological features of DCs mediated by SMMC-7721 cells [145] (Figure 1, Table 3).

CD4+ CD25+ regulatory T cells (Tregs) originate from CD4+ Th0 cells upon stimulation of TGF-β and Foxp3 expression. Tregs may produce IL-10 and function as a negative immune regulator. Astragalus (Huang-Qi) polysaccharides (APS) inhibits Foxp3 expression and proliferation of CD4+ CD25+ Treg cells. APS also inhibits Treg cell migration by blocking SDF-1 or its receptor via CXCR4/CXCL12 pathway [146]. *Radix Glycyrrhizae* (Gan-Cao) polysaccharide down-regulates Tregs, related cytokines IL-10 and TGF-β, and Foxp3 expression, and increases IL-2 and IL-12p70 level in serum in H22 HCC-bearing mice [147] (Figure 1, Table 3).

**Table 3:** Effects of herbal compounds on immune function and angiogenesis in hepatocarcinoma.

Compounds	Herbs	Effects	Targets/Molecular Events	Ref.
*Lycium barbarum* polysaccharide	*Lycium barbarum* (Gou-Qi)	↑ CD4+ and CD8+ T cells in H22 hepatoma; promote dendritic cells to stimulate allogeneic lymphocyte proliferation, produce IL-12p70 and IFN-γ	Unknown; NF-κB	[138,144]
Polysaccharides	*Artemisia annua* L. (Huang-Hua-Hao)	↑ CD4+ and CD8+ T cells, IFN-γ and IL-4 secretion, and induce cancer cell apoptosis in human hepatoma 7402 bearing mice	Unknown	[139]
Gastrodin	*Gastrodia elata* Blume (Tian-Ma)	↑ cytotoxic activities of NK and CD8+ T cells against H22 cells	↑ NF-κB, IL-2 and Bcl-2 in CD4+ T cells	[140]
Shikonin	*Lithospermum erythrorhizon* (Zi-Cao)	↑ CD3+ and CD19+ lymphocytes, NK activities and IL-2 in HepA22 bearing mice	Unknown	[141]
Proteins extract	*Omphalia lapidesces* (Lei-Wan)	↑ spleen mass and IFN-γ production in H22 hepatocarcinoma bearing mice	Unknown	[142]
*Gekko* sulfated polysaccharide-protein complex	*Gekko swinhonis Guenther* (Tian-Long)	Restore the defective biorheological characteristics of dendritic cells mediated by SMMC-7721 cells	Unknown	[145]
*Astragalus* polysaccharides	*Astragalus membranaceous* (Huang-Qi)	↓ proliferation and migration in CD4+ CD25+ Treg cells	↓ Foxp3, SDF-1 or its receptor through the CXCR4/CXCL12 pathway	[146]
Polysaccharide	*Radix Glycyrrhizae* (Gan-Cao)	↓ Tregs cells	↓ Foxp3	[147]
*Gekko*-sulfated glycopeptide	*Gekko swinhonis Guenther* (Tian-Long)	↓ bFGF stimulated proliferation and migration of endothelial cells, angiogenesis and tumor growth in liver cancer	↓ bFGF secretion and binding to heparin/heparan sulfate	[148]
Pedicularioside G	*Pedicularis striata* (Ma-Xian-Hao)	↓ proliferation and migration in HUVEC cells, and angiogenesis in chicken embryo chorioallantoic membrane and hepatoma	↓ reactive oxygen species	[149]
Vitexin compound 1	*Vitex negundo* (Huang-Jing)	↓ proliferation and cell cycle arrest at G1/G0 in hepatocellular carcinoma cells, and HUVEC tube formation	↓ VEGF	[150]
Resveratrol	*Polygonum cuspidatum* (Hu*-*Zhang), *etc.*	↓ proliferation in liver cancer cells	↓ hypoxia-induced activation of ERK1/2 and Akt, HIF-1α and VEGF expression	[151,152]
Cinobufotalin, Panax notoginseng saponins, Ginsenosides Rg3 and Lentinan	*Bufonis Venenum* (Chan-Su), *Panax notoginseng* (San-Qi), *P. ginseng* (Ren-Shen), *Lentinula edodes* (Xiang-Gu)	↓ angiogenesis and tumor growth in H22 hepatocellular carcinoma	↓ VEGF, EGFR and MMP-2 expression	[153]

↓ Inhibit or down-regulate, ↑ promote or up-regulate.

## 13. Inhibition of Angiogenesis

Angiogenesis, the process of new blood vessels generating from existing vessels, plays a crucial role in tumor growth and metastasis and has been recognized as a potential target for HCC treatment [154,155]. Some Chinese herbal components have demonstrated anti-angiogenic effects.

*G. chinensis* (Tian-Long) inhibits tumor growth, induces apoptosis, and inhibits angiogenesis accompanied by down-regulation of VEGF and bFGF in H22 HCC [156]. Gekko-sulfated glycopeptide decreases bFGF secretion and binding to its low affinity receptor heparin/heparan sulfate, inhibits bFGF stimulated proliferation and migration of endothelial cells, and thus inhibits angiogenesis and tumor growth in liver cancer [148] (Figure 1, Table 3).

Pedicularioside G, a phenylpropanoid glycoside isolated from *Pedicularis striata* (Ma-Xian-Hao), inhibits proliferation and migration in human umbilical vein endothelial cells (HUVEC), and angiogenesis in chicken embryo chorioallantoic membrane and human hepatoma. Pedicularioside G also inhibited cell proliferation and migration and tumor growth in human hepatoma. The effects of pedicularioside G are partially related to down-regulation of ROS [149] (Figure 1, Table 3).

Vitexin compound 1, a compound isolated from *V. negundo* (Huang-Jing), inhibits cell proliferation and arrests cell cycle at G1/G0 in HCC cells. Vitexin compound 1 also inhibited VEGF secretion and HUVEC tube formation [150] (Figure 1). Resveratrol, a compound that can be isolated from *P. cuspidatum* (Hu-Zhang), may inhibit proliferation and VEGF expression in HepG2 hepatoma cells [151]. Resveratrol also reduces hypoxia induced accumulation of hypoxia inducible factor-1α and VEGF expression in hepatoma HepG2 cells [152] (Table 3).

QHF, a herbal component formula composed of cinobufotalin, *Panax notoginseng* saponins, ginsenosides Rg3 and lentinan, inhibits VEGF, EGFR and MMP-2 expression, as well as angiogenesis and tumor growth in H22 HCC [153] (Table 3).

## 14. Herbal Compound-Based Combinational Treatment

Chinese herbs are usually prescribed as formulas guided by TCM theories, *i.e.*, combining multiple herbs in a prescription [157]. It is rational to use herbal components in the same principle.

*C. kwangsiensis* or *C. phaeocaulis* or *C. wenyujin* or *C. longa* (Yu-Jin) and *P. cuspidatum* (Hu-Zhang) are frequently used simultaneously as herbal-pairs in liver cancer treatment based on complementary traditional efficacy. Curcumin is a component of Yu-Jin and resveratrol is a compound can be isolated from Hu-Zhang. We have found that curcumin combined with resveratrol may synergistically inhibit XIAP (X-linked inhibitor of apoptosis protein) and survivin expression, up-regulate ROS production, and activate caspase-3, -8 and -9 to induce apoptosis in HCC cells [158] (Table 4).

Based on TCM principles, Chen *et al.* have established a herbal components formula composed of cinobufotalin, cantharidin, *Panax notoginseng* saponins (PNS), tanshinone, ginsenosides Rg3 and lentinan. These compounds are derived from toad skin (Gan-Chan-Pi), *Mylabris phalerata* Pallas or *M. cichorii* Linnaeus (Ban-Mao), *Panax notoginseng* (San-Qi), *S. miltiorrhiza* Bunge (Dan-Shen), *P. ginseng* (Ren-Shen) and *Lentinula edodes* (Xiang-Gu), respectively. The formula has demonstrated efficacy in inhibiting tumor growth, prolonging survival time, enhancing anticancer effects and reducing toxicity of cisplatin in HCC bearing mice [159] (Table 4).

In addition to components from different herbs, different components from the same herb can also be used synchronously for liver cancer treatment. Acetylshikonin and β,β-dimethylacrylshikonin, components of *L. erythrorhizon* (Zi-Cao), exhibit anticancer activity against HCC [160,161]. Aikete injection, a mixture composed of acetylshikonin and β,β-dimethyl-acrylshikonin, inhibits proliferation, induces apoptosis, and arrests cell cycle at the G2/M phase accompanied by down-regulation of Bcl-2 and Bcl-2/Bax ratio in HCC cells [162] (Table 4).

**Table 4:** Herbal compound-based combinational treatment.

Compounds	Herbs	Effects	Targets/Molecular Events	Ref.
Astragalosides, Astragalus polysaccharide and salvianolic acids	*Astragalus membranaceous* (Huang-Qi), *Salvia miltiorrhiza* Bunge (Dan-shen)	↓ DEN induced hepatocarcinogenesis; ↓ TGF-β1-induced cell invasion in HepG2 cells	↓ GST-P and α-SMA; modulating TGF-β/Smad signaling	[13,109]
Cinobufotalin, Panax notoginseng saponins, Ginsenosides Rg3 and Lentinan	*Bufonis Venenum* (Chan-Su), *Panax notoginseng* (San-Qi), *Panax ginseng* (Ren-Shen), *Lentinula edodes* (Xiang-Gu)	↓ angiogenesis and tumor growth in H22 hepatocellular carcinoma	↓ VEGF, EGFR and MMP-2 expression	[153]
Curcumin and resveratrol	*Curcuma kwangsiensis* (Yu-Jin or Er-Zhu) or *C. phaeocaulis* (Yu-Jin or Er-Zhu) or *C. wenyujin* (Yu-Jin or Er-Zhu) or *C. longa* (Yu-Jin or Jiang-Huang), and *Polygonum cuspidatum* (Hu-Zhang), *etc.*	↓ proliferation, ↑ apoptosis in Hepa1-6 cells	↓ XIAP and Survivin, ↑ ROS production, caspase-3, -8 and -9	[158]
Cinobufotalin, Cantharidin, Panax notoginseng saponins, Tanshinone, Ginsenosides Rg3 and Lentinan	*Bufonis Venenum* (Chan-Su), *Mylabris phalerata* Pallas or *M. cichorii* Linnaeus (Ban-Mao), *Panax notoginseng* (San-Qi), *Salvia miltiorrhiza* Bunge (Dan-Shen), *Panax ginseng* (Ren-Shen), *Lentinula edodes* (Xiang-Gu)	Inhibit tumor growth, prolong survival time, enhance anticancer effects and reduce toxicity of cisplatin in hepatocellular carcinoma bearing mice	Unknown	[159]
Acetylshikonin and β,β-dimethylacrylshikonin	*Lithospermum erythrorhizon* (Zi-Cao)	↓ proliferation, ↑ apoptosis, arrest cell cycle in G2/M phase in SMMC-7721 cells	↓ Bcl-2 and Bcl-2/Bax ratio	[162]

↓ Inhibit or down-regulate, ↑ promote or up-regulate.

## 15. Conclusions and Future Directions

In summary, Chinese herbal compounds have demonstrated multiple effects against HCC, including prevention of hepatocarcinogenesis, inhibition of cell proliferation, induction of apoptosis, autophagy, cell senescence and anoikis, cell cycle arrest, inhibition of EMT, metastasis and angiogenesis, regulation of immune function, reversal of drug resistance and enhancement of chemotherapeutic effects (Figure 1). These observations provide the basis for further development of new drugs for HCC management.

Chinese herbs are natural products and most herbs are safe for human consumption. One herb may contain multiple anticancer compounds, e.g., *C. wenyujin* (Er-Zhu or Yu-Jin) contains curcumin, curcumol and β-elemene. One herbal compound may have multiple effects on HCC, such as curcumin, which has demonstrated effects in inhibiting hepatocarcinogenesis and cell proliferation, inducing apoptosis and autophagy, and inhibiting EMT in HCC cells via multiple targets [8,78,79,80].

Chinese herbs have a long history of clinical use. Thousands years of clinical experiences provide clues for pharmacological studies. Compounds in some clinically-used anticancer herbs have not yet been fully identified, such as *S. lyratum* Thunberg (Bai-Ying) and *Patrinia scabra* Bunge (Mu-Tou-Hui). Whether compounds from these herbs possess more potent anticancer effects on HCC are worthy of further study.

Some anticancer compounds containing herbs are neither commonly clinically used nor recognized as anticancer herbs so far, for example, *A. pusilla* (Jiu-Jie-Long) [21] and *P. capitatum* (Tou-Hua-Liao) [22]. Whether these herbs possess anticancer effects on HCC warrants more investigation.

Some herbal compounds shown significant anticancer effects in HCC, but the corresponding herbs are not clinically used as anticancer herbs or their anticancer effects have not been observed so far, such as *A. dahurica* (Bai-Zhi) [117], *M. officinalis* (Hou-Po) [48,49] and *A. officinarum* Hance (Gao-Liang-Jiang) [92]. The different effects between herb and herbal components may result from relatively insufficient quantities of anticancer compounds in those herbs. 

Combinational treatment with multiple herbal compounds is a promising strategy for application of herbal compounds. The combinational principles or compatibility of Chinese herbs provide a theoretical basis for combinational treatment with multiple herbal compounds for HCC. In addition, Chinese herbal formulas or known active herbal-pairs provide clues for the extraction of compounds from multiple herbs and combinational treatment with these compounds.

Current treatments for HCC are less than satisfactory. Anticancer herbal compounds are importent resources for the development of new drugs for liver cancer treatment. These compounds can be developed as single herbal compound drugs with or without chemical modifications, or in combination with other herbal compounds, herbs and even modern drugs guided by the principles of TCM and/or pharmacological interaction.

## References

[1] Forner A., Llovet J.M., Bruix J. (2012). Hepatocellular carcinoma. Lancet.

[2] Tang Z.Y. (2011). Combination of traditional Chinese medicine and western medicine in the treatment of liver cancer. J. Clin. Hepatol..

[3] Wu M.C. (2003). Traditional Chinese medicine in prevention and treatment of liver cancer: Function, status and existed problems. J. Chin. Integr. Med..

[4] Hu B., Wang S.S., Du Q. (2015). Traditional Chinese Medicine for the prevention and treatment of hepatocarcinoma: From bench to bedside. World J. Hepatol..

[5] Mao W.C., Song Y.J., Zhang J., Jiao Y.B., Feng L.L., Bai H.F., Wang X.P., Cai D.Y., Wang Y.Q. (2012). Effect of ursolic acid on DEN-induced hepatic precancerous lesions in mice. Chin. J. Integr. Tradit. West. Med. Liver Dis..

[6] Gayathri R., Priya D.K., Gunassekaran G.R., Sakthisekaran D. (2009). Ursolic acid attenuates oxidative stress-mediated hepatocellular carcinoma induction by diethylnitrosamine in male Wistar rats. Asian Pac. J. Cancer Prev..

[7] Lin Y.L., Hsu J.D., Chou F.P., Lee M.J., Shiow S.J., Wang C.J. (2000). Suppressive effect of penta-acetyl geniposide on the development of γ-glutamyl transpeptidase foci-induced by aflatoxin B_1_ in rats. Chem. Biol. Interact..

[8] Chuang S.E., Kuo M.L., Hsu C.H., Chen C.R., Lin J.K., Lai G.M., Hsieh C.Y., Cheng A.L. (2000). Curcumin-containing diet inhibits diethylnitrosamine-induced murine hepatocarcinogenesis. Carcinogenesis.

[9] Zhao X., Zhang J.J., Wang X., Bu X.Y., Lou Y.Q., Zhang G.L. (2008). Effect of berberine on hepatocyte proliferation, inducible nitric oxide synthase expression, cytochrome P450 2E1 and 1A2 activities in diethylnitrosamine- and phenobarbital-treated rats. Biomed. Pharmacother..

[10] Lu X.L., He S.X., Ren M.D., Wang Y.L., Zhang Y.X., Liu E.Q. (2012). Chemopreventive effect of saikosaponin-d on diethylinitrosamine-induced hepatocarcinogenesis: Involvement of CCAAT/enhancer binding protein β and cyclooxygenase-2. Mol. Med. Rep..

[11] Nomura M., Nakachiyama M., Hida T., Ohtaki Y., Sudo K., Aizawa T., Aburada M., Miyamoto K.I. (1994). Gomisin A, a lignan component of *Schizandora* fruits, inhibits development of preneoplastic lesions in rat liver by 3′-methyl-4-dimethylamino-azobenzene. Cancer Lett..

[12] Jia X., Han C., Chen J. (2001). Studies on the inhibitory effects of tea polyphenols and tea pigments on liver precancerous lesion in rats. J. Hyg. Res..

[13] Rui W., Xie L., Liu X., He S., Wu C., Zhang X., Zhang L., Yang Y. (2014). Compound *Astragalus* and *Salvia miltiorrhiza* extract suppresses hepatocellular carcinoma progression by inhibiting fibrosis and PAI-1 mRNA transcription. J. Ethnopharmacol..

[14] Yan R.Q., Chen Z.Y., Qin G.Z., Qin L.L. (1986). Effects of twelve herbs on Aflatoxin B1 induced hepatocarcinogensis in rats. J. Guangxi Med. Coll..

[15] Ruan C.C., Liang Y., Liu Z.H. (1987). Inhibition of 12 Chinese Traditional Medicinal herbs on mutagenic effects induced by Aflatoxin B1. Chin. J. Cancer.

[16] Wang Q.L., Wu Q., Tao Y.Y., Liu C.H., El-Nezami H. (2011). Salvianolic acid B modulates the expression of drug-metabolizing enzymes in HepG2 cells. Hepatobiliary Pancreat. Dis. Int..

[17] Liu H., Chou G.X., Wang J.M., Ji L.L., Wang Z.T. (2011). Steroidal saponins from the rhizomes of *Dioscorea bulbifera* and their cytotoxic activity. Planta Med..

[18] Wang Y., Ma J., Chow S.C., Li C.H., Xiao Z., Feng R., Fu J., Chen Y. (2014). A potential antitumor ellagitannin, davidiin, inhibited hepatocellular tumor growth by targeting EZH2. Tumour. Biol..

[19] Bao F., Qiu J., Zhang H. (2012). Potential role of β-elemene on histone H1 in the H22 ascites hepatoma cell line. Mol. Med. Rep..

[20] Tao X., Wang P., Yang X., Yao H., Liu J., Cao Y. (2005). Inhibitory effect of ardipusilloside-I on Lewis pulmonary carcinoma and hepatocarcinoma SMMC-7721. J. Chin. Med. Mater..

[21] Wang M.K., Ding L.S., Wu F.E. (2008). Antitumor effects of raddeanin A on S180, H22 and U14 cell xenografts in mice. Chin. J. Cancer.

[22] Wu Y., Zhang Z.X., Hu H., Li D., Qiu G., Hu X., He X. (2011). Novel indole C-glycosides from *Isatis indigotica* and their potential cytotoxic activity. Fitoterapia.

[23] Lu Q., Jiang M.H., Jiang J.G., Zhang R.F., Zhang M.W. (2012). Isolation and identification of compounds from *Penthorum chinense* Pursh with antioxidant and antihepatocarcinoma properties. J. Agric. Food Chem..

[24] Chen Y., Xu S.S., Chen J.W., Wang Y., Xu H.Q., Fan N.B., Li X. (2012). Anti-tumor activity of *Annona squamosa* seeds extract containing annonaceous acetogenin compounds. J. Ethnopharmacol..

[25] Huang J., Tang X.H., Ikejima T., Sun X.J., Wang X.B., Xi R.G., Wu L.J. (2008). A new triterpenoid from *Panax ginseng* exhibits cytotoxicity through p53 and the caspase signaling pathway in the HepG2 cell line. Arch. Pharm. Res..

[26] Li X., Guan Y., Zhou X., Sun L., Liu Y., He Q., Fu L., Mao Y.Q. (2005). Anticarcinogenic effect of 20(*R*)-ginsenoside Rg3 on induced hepatocellular carcinoma in rats. J. Sichuan Univ. Med. Sci. Edit..

[27] Chen J.C., Chung J.G., Chen L.D. (1999). Gypenoside induces apoptosis in human Hep3B and HA22T tumour cells. Cytobios..

[28] Teng B.S., Lu Y.H., Wang Z.T., Tao X.Y., Wei D.Z. (2006). *In vitro* anti-tumor activity of isorhamnetin isolated from *Hippophae rhamnoides* L. against BEL-7402 cells. Pharmacol. Res..

[29] Zhou M., Higo H., Cai Y. (2010). Inhibition of hepatoma 22 tumor by Liquiritigenin. Phytother. Res..

[30] Lou L., Ye W., Chen Y., Wu S., Jin L., He J., Tao X., Zhu J., Chen X., Deng A., Wang J. (2012). Ardipusilloside inhibits survival, invasion and metastasis of human hepatocellular carcinoma cells. Phytomedicine.

[31] Chen Y.L., Jian M.H., Lin C.C., Kang J.C., Chen S.P., Lin P.C., Hung P.J., Chen J.R., Chang W.L., Lin S.Z. (2008). The induction of orphan nuclear receptor Nur77 expression by *n*-butylenephthalide as pharmaceuticals on hepatocellular carcinoma cell therapy. Mol. Pharmacol..

[32] Zhang M., Chen H., Huang J., Li Z., Zhu C., Zhang S. (2005). Effect of *lycium barbarum* polysaccharide on human hepatoma QGY7703 cells: Inhibition of proliferation and induction of apoptosis. Life Sci..

[33] Cai J., Zhao X.L., Liu A.W., Nian H., Zhang S.H. (2011). Apigenin inhibits hepatoma cell growth through alteration of gene expression patterns. Phytomedicine.

[34] Li S., Dong P., Wang J., Zhang J., Gu J., Wu X., Wu W., Fei X., Zhang Z., Wang Y. (2010). Icariin, a natural flavonol glycoside, induces apoptosis in human hepatoma SMMC-7721 cells via a ROS/JNK-dependent mitochondrial pathway. Cancer Lett..

[35] He J., Wang Y., Duan F., Jiang H., Chen M.F., Tang S.Y. (2010). Icaritin induces apoptosis of HepG2 cells via the JNK1 signaling pathway independent of the estrogen receptor. Planta Med..

[36] Song G., Luo Q., Qin J., Wang L., Shi Y., Sun C. (2006). Effects of oxymatrine on proliferation and apoptosis in human hepatoma cells. Colloids Surf. B Biointerfaces.

[37] Xu H., Zhang S. (2013). Scutellarin-induced apoptosis in HepG2 hepatocellular carcinoma cells via a STAT3 pathway. Phytother. Res..

[38] Bao W., Pan H., Lu M., Ni Y., Zhang R., Gong X. (2007). The apoptotic effect of sarsasapogenin from *Anemarrhena asphodeloides* on HepG2 human hepatoma cells. Cell Biol. Int..

[39] Chan J.Y., Tang P.M., Hon P.M., Au S.W., Tsui S.K., Waye M.M., Kong S.K., Mak T.C., Fung K.P. (2006). Pheophorbide a, a major antitumor component purified from *Scutellaria barbata*, induces apoptosis in human hepatocellular carcinoma cells. Planta Med..

[40] Ding X., Zhu F.S., Li M., Gao S.G. (2012). Induction of apoptosis in human hepatoma SMMC-7721 cells by solamargine from *Solanum nigrum* L. J. Ethnopharmacol..

[41] Zhang J.F., Liu P.Q., Chen G.H., Lu M.Q., Cai C.J., Yang Y., Li H. (2007). Ponicidin inhibits cell growth on hepatocellular carcinoma cells by induction of apoptosis. Dig. Liver Dis..

[42] Sun G.P., Wang H., Xu S.P., Shen Y.X., Wu Q., Chen Z.D., Wei W. (2008). Anti-tumor effects of paeonol in a HepA-hepatoma bearing mouse model via induction of tumor cell apoptosis and stimulation of IL-2 and TNF-alpha production. Eur. J. Pharmacol..

[43] Lee W.Y., Liu K.W., Yeung J.H. (2009). Reactive oxygen species-mediated kinase activation by dihydrotanshinone in tanshinones-induced apoptosis in HepG2 cells. Cancer Lett..

[44] Xie Q., Yang Y., Wang Z., Chen F., Zhang A., Liu C. (2014). Resveratrol-4-O-d-(2′-galloyl)-glucopyranoside isolated from *Polygonum cuspidatum* exhibits anti-hepatocellular carcinoma viability by inducing apoptosis via the JNK and ERK pathway. Molecules.

[45] Wang Y., Deng L., Zhong H., Wang Y., Jiang X., Chen J. (2011). Natural plant extract tubeimoside I promotes apoptosis-mediated cell death in cultured human hepatoma (HepG2) cells. Biol. Pharm. Bull..

[46] Chang C., Zhu Y.Q., Mei J.J., Liu S.Q., Luo J. (2010). Involvement of mitochondrial pathway in NCTD-induced cytotoxicity in human hepG2 cells. J. Exp. Clin. Cancer Res..

[47] He Y., Wang J., Liu X., Zhang L., Yi G., Li C., He X., Wang P., Jiang H. (2010). Toosendanin inhibits hepatocellular carcinoma cells by inducing mitochondria-dependent apoptosis. Planta Med..

[48] Deng J., Qian Y., Geng L., Chen J., Wang X., Xie H., Yan S., Jiang G., Zhou L., Zheng S. (2008). Involvement of p38 mitogen-activated protein kinase pathway in honokiol-induced apoptosis in a human hepatoma cell line (hepG2). Liver Int..

[49] Lin S.Y., Chang Y.T., Liu J.D., Yu C.H., Ho Y.S., Lee Y.H., Lee W.S. (2001). Molecular mechanisms of apoptosis induced by magnolol in colon and liver cancer cells. Mol. Carcinog..

[50] Yan S.L., Huang C.Y., Wu S.T., Yin M.C. (2010). Oleanolic acid and ursolic acid induce apoptosis in four human liver cancer cell lines. Toxicol. in Vitro.

[51] Lu C.C., Yang J.S., Huang A.C., Hsia T.C., Chou S.T., Kuo C.L., Lu H.F., Lee T.H., Wood W.G., Chung J.G. (2010). Chrysophanol induces necrosis through the production of ROS and alteration of ATP levels in J5 human liver cancer cells. Mol. Nutr. Food Res..

[52] Du Q., Bian X.L., Xu X.L., Zhu B., Yu B., Zhai Q. (2013). Role of mitochondrial permeability transition in human hepatocellular carcinoma Hep-G2 cell death induced by rhein. Fitoterapia.

[53] Shi P., Huang Z., Chen G. (2008). Rhein induces apoptosis and cell cycle arrest in human hepatocellular carcinoma BEL-7402 cells. Am. J. Chin. Med..

[54] Wang J.G., Zheng X.X., Zeng G.Y., Zhou Y.J., Yuan H. (2014). Purified vitexin compound 1 induces apoptosis through activation of FOXO3a in hepatocellular carcinoma. Oncol. Rep..

[55] Chang Y.F., Chi C.W., Wang J.J. (2006). Reactive oxygen species production is involved in quercetin-induced apoptosis in human hepatoma cells. Nutr. Cancer.

[56] Yang Y., Yang L., You Q.D., Nie F.F., Gu H.Y., Zhao L., Wang X.T., Guo Q.L. (2007). Differential apoptotic induction of gambogic acid, a novel anticancer natural product, on hepatoma cells and normal hepatocytes. Cancer Lett..

[57] Bao Y.R., Wang S., Meng X.S., Chou J., Yang X.X. (2013). Effect of flavonoids of *polygoni orientails* fructus on human hepatoma cell line SMMC-7721. J. Chin. Med. Mater..

[58] Zhao C.Y., Hui L.P., He L., Li Q. (2013). Study on inhibitory effect of triterpenoid saponin from *Ardisia japonica* TSP02 on proliferation and metastasis of human hepatocellular carcinoma cells and its mechanism. Chin. J. Chin. Mater. Med..

[59] Xie R.F., Li Z.C., Gao B., Shi Z.N., Zhou X. (2012). Bufothionine, a possible effective component in cinobufocini injection for hepatocellular carcinoma. J. Ethnopharmacol..

[60] Wang H., Ye Y., Chui J.H., Zhu G.Y., Li Y.W., Fong D.W., Yu Z.L. (2010). Oridonin induces G2/M cell cycle arrest and apoptosis through MAPK and p53 signaling pathways in HepG2 cells. Oncol. Rep..

[61] Huang L.Z., Wang J., Lu F.T., Yang F.C., Chen X., Hong X., Jiang X.S. (2013). Mechanism study on anti-proliferative effects of curcumol in human hepatocarcinoma HepG2 cells. Chin. J. Chin. Mater. Med..

[62] Hsu Y.L., Kuo P.L., Chiang L.C., Lin C.C. (2004). Involvement of p53, nuclear factor kappaB and Fas/Fas ligand in induction of apoptosis and cell cycle arrest by saikosaponin d in human hepatoma cell lines. Cancer Lett..

[63] Zhang Z., Duan C., Ding K., Wang Z. (2009). WT inhibit human hepatocellular carcinoma BEL-7402 cells growth by modulating Akt and ERK1/2 phosphorylation. Chin. J. Chin. Mater. Med..

[64] Ma X., Jin S., Zhang Y., Wan L., Zhao Y., Zhou L. (2014). Inhibitory effects of nobiletin on hepatocellular carcinoma *in vitro* and *in vivo*. Phytother. Res..

[65] Ma L., Wen S., Zhan Y., He Y., Liu X., Jiang J. (2008). Anticancer effects of the Chinese medicine matrine on murine hepatocellular carcinoma cells. Planta Med..

[66] Zhang J.Q., Li Y.M., Liu T., He W.T., Chen Y.T., Chen X.H., Li X., Zhou W.C., Yi J.F., Ren Z.J. (2010). Antitumor effect of matrine in human hepatoma G2 cells by inducing apoptosis and autophagy. World J. Gastroenterol..

[67] Yu H.B., Zhang H.F., Li D.Y., Zhang X., Xue H.Z., Zhao S.H. (2011). Matrine inhibits matrix metalloproteinase-9 expression and invasion of human hepatocellular carcinoma cells. J. Asian Nat. Prod. Res..

[68] Yip N.K., Ho W.S. (2013). Berberine induces apoptosis via the mitochondrial pathway in liver cancer cells. Oncol. Rep..

[69] Yu R., Zhang Z.Q., Wang B., Jiang H.X., Cheng L., Shen L.M. (2014). Berberine-induced apoptotic and autophagic death of HepG2 cells requires AMPK activation. Cancer Cell Int..

[70] Kuo H.M., Tsai H.C., Lin Y.L., Yang J.S., Huang A.C., Yang M.D., Hsu S.C., Chung M.C., Gibson Wood W., Chung J.G. (2009). Mitochondrial-dependent caspase activation pathway is involved in baicalein-induced apoptosis in human hepatoma J5 cells. Int. J. Oncol..

[71] Zhang X., Tang X., Liu H., Li L., Hou Q., Gao J. (2012). Autophagy induced by baicalin involves downregulation of CD147 in SMMC-7721 cells *in vitro*. Oncol. Rep..

[72] Liu W., Mu R., Nie F.F., Yang Y., Wang J., Dai Q.S., Lu N., Qi Q., Rong J.J., Hu R. (2009). MAC-related mitochondrial pathway in oroxylin-A-induced apoptosis in human hepatocellular carcinoma HepG2 cells. Cancer Lett..

[73] Zou M., Lu N., Hu C., Liu W., Sun Y., Wang X., You Q., Gu C., Xi T., Guo Q. (2012). Beclin 1-mediated autophagy in hepatocellular carcinoma cells: Implication in anticancer efficiency of oroxylin A via inhibition of mTOR signaling. Cell Signal..

[74] Zhu B., Zhao L., Zhu L., Wang H., Sha Y., Yao J., Li Z., You Q., Guo Q. (2012). Oroxylin A reverses CAM-DR of HepG2 cells by suppressing Integrinβ1 and its related pathway. Toxicol. Appl. Pharmacol..

[75] Gong K., Li W. (2011). Shikonin, a Chinese plant-derived naphthoquinone, induces apoptosis in hepatocellular carcinoma cells through reactive oxygen species: A potential new treatment for hepatocellular carcinoma. Free Radic. Biol. Med..

[76] Gong K., Zhang Z., Chen Y., Shu H.B., Li W. (2014). Extracellular signal-regulated kinase, receptor interacting protein, and reactive oxygen species regulate shikonin-induced autophagy in human hepatocellular carcinoma. Eur. J. Pharmacol..

[77] Wei P.L., Tu C.C., Chen C.H., Ho Y.S., Wu C.T., Su H.Y., Chen W.Y., Liu J.J., Chang Y.J. (2013). Shikonin suppresses the migratory ability of hepatocellular carcinoma cells. J. Agric. Food Chem..

[78] Wang W.Z., Li L., Liu M.Y., Jin X.B., Mao J.W., Pu Q.H., Meng M.J., Chen X.G., Zhu J.Y. (2013). Curcumin induces FasL-related apoptosis through p38 activation in human hepatocellular carcinoma Huh7 cells. Life Sci..

[79] Qian H., Yang Y., Wang X. (2011). Curcumin enhanced adriamycin-induced human liver-derived Hepatoma G2 cell death through activation of mitochondria-mediated apoptosis and autophagy. Eur. J. Pharm. Sci..

[80] Chang Y.H., Jiang M., Liu K.G., Li X.Q. (2013). Curcumin inhibited hypoxia induced epithelial-mesenchymal transition in hepatic carcinoma cell line HepG2 *in vitro*. Chin. J. Integr. Tradit. West. Med..

[81] Du Q., Shen K.P., Hu B., Deng S. (2012). Effects of resveratrol on apoptosis and ROS production in Hepa 1–6 hepatocarcinoma cells. J. Chin. Med. Mater..

[82] Liao P.C., Ng L.T., Lin L.T., Richardson C.D., Wang G.H., Lin C.C. (2010). Resveratrol arrests cell cycle and induces apoptosis in human hepatocellular carcinoma Huh-7 cells. J. Med. Food..

[83] Du Q., Chen Y.L. (2007). The effects of Resveratrol on proliferation, apoptosis and invasion of Bel-7404 hepatocarcinoma cell line. J. Fujian Med. Univ..

[84] Yu H.B., Pan C.E., Wu W.J., Zhao S.H., Zhang H.F. (2008). Effects of resveratrol on matrix metalloproteinase-9 expression in hepatoma cells. J. Chin. Integr. Med..

[85] Qi F., Inagaki Y., Gao B., Cui X., Xu H., Kokudo N., Li A., Tang W. (2011). Bufalin and cinobufagin induce apoptosis of human hepatocellular carcinoma cells via Fas- and mitochondria-mediated pathways. Cancer Sci..

[86] Miao Q., Bi L.L., Li X., Miao S., Zhang J., Zhang S., Yang Q., Xie Y.H., Zhang J., Wang S.W. (2013). Anticancer effects of Bufalin on human hepatocellular carcinoma HepG2 cells: Roles of apoptosis and autophagy. Int. J. Mol. Sci..

[87] Liu C., Gong K., Mao X., Li W. (2011). Tetrandrine induces apoptosis by activating reactive oxygen species and repressing Akt activity in human hepatocellular carcinoma. Int. J. Cancer.

[88] Ng L.T., Chiang L.C., Lin Y.T., Lin C.C. (2006). Antiproliferative and apoptotic effects of tetrandrine on different human hepatoma cell lines. Am. J. Chin. Med..

[89] Gong K., Chen C., Zhan Y., Chen Y., Huang Z., Li W. (2012). Autophagy-related gene 7 (ATG7) and reactive oxygen species/extracellular signal-regulated kinase regulate tetrandrine-induced autophagy in human hepatocellular carcinoma. J. Biol. Chem..

[90] Zhang D.M., Liu J.S., Deng L.J., Chen M.F., Yiu A., Cao H.H., Tian H.Y., Fung K.P., Kurihara H., Pan J.X. (2013). Arenobufagin, a natural bufadienolide from toad venom, induces apoptosis and autophagy in human hepatocellular carcinoma cells through inhibition of PI3K/Akt/mTOR pathway. Carcinogenesis.

[91] Chu Y.L., Ho C.T., Chung J.G., Rajasekaran R., Sheen L.Y. (2012). Allicin induces p53-mediated autophagy in Hep G2 human liver cancer cells. J. Agric. Food Chem..

[92] Wen M., Wu J., Luo H., Zhang H. (2012). Galangin induces autophagy through upregulation of p53 in HepG2 cells. Pharmacology.

[93] Huang W.W., Tsai S.C., Peng S.F., Lin M.W., Chiang J.H., Chiu Y.J., Fushiya S., Tseng M.T., Yang J.S. (2013). Kaempferol induces autophagy through AMPK and AKT signaling molecules and causes G2/M arrest via downregulation of CDK1/cyclin B in SK-HEP-1 human hepatic cancer cells. Int. J. Oncol..

[94] Chen L., Ye H.L., Zhang G., Yao W.M., Chen X.Z., Zhang F.C., Liang G. (2014). Autophagy inhibition contributes to the synergistic interaction between EGCG and doxorubicin to kill the hepatoma Hep3B cells. PLoS ONE.

[95] Lin Y., Wang K., Hu C., Lin L., Qin S., Cai X. (2014). Elemene injection induced autophagy protects human hepatoma cancer cells from starvation and undergoing apoptosis. Evid. Based Complement. Altern. Med..

[96] Chang U.M., Li C.H., Lin L.I., Huang C.P., Kan L.S., Lin S.B. (2006). Ganoderiol F, a ganoderma triterpene, induces senescence in hepatoma HepG2 cells. Life Sci..

[97] Cheng H.L., Su S.J., Huang L.W., Hsieh B.S., Hu Y.C., Hung T.C., Chang K.L. (2010). Arecoline induces HA22T/VGH hepatoma cells to undergo anoikis - involvement of STAT3 and RhoA activation. Mol. Cancer.

[98] Yuan S.L., Wei Y.Q., Wang X.J., Xiao F., Li S.F., Zhang J. (2004). Growth inhibition and apoptosis induction of tanshinone II-A on human hepatocellular carcinoma cells. World J. Gastroenterol..

[99] Wang W.Q., Liu L., Sun H.C., Fu Y.L., Xu H.X., Chai Z.T., Zhang Q.B., Kong L.Q., Zhu X.D., Lu L. (2012). Tanshinone IIA inhibits metastasis after palliative resection of hepatocellular carcinoma and prolongs survival in part via vascular normalization. J. Hematol. Oncol..

[100] Yu X.X., Feng T., Ren L., Zheng C.L. (2009). Tanshinone II-A inhibits invasion and metastasis of human hepatocellular carcinoma cells *in vitro* and *in vivo*. Tumori..

[101] Zhang C.Z., Zhang H., Yun J., Chen G.G., Lai P.B. (2012). Dihydroartemisinin exhibits antitumor activity toward hepatocellular carcinoma *in vitro* and *in vivo*. Biochem. Pharmacol..

[102] Weifeng T., Feng S., Xiangji L., Changqing S., Zhiquan Q., Huazhong Z., Peining Y., Yong Y., Mengchao W., Xiaoqing J. (2011). Artemisinin inhibits *in vitro* and *in vivo* invasion and metastasis of human hepatocellular carcinoma cells. Phytomedicine.

[103] Yao W.L., Ko B.S., Liu T.A., Liang S.M., Liu C.C., Lu Y.J., Tzean S.S., Shen T.L., Liou J.Y. (2014). Cordycepin suppresses integrin/FAK signaling and epithelial-mesenchymal transition in hepatocellular carcinoma. Anticancer Agents Med. Chem..

[104] Zheng J., Li C., Wu X., Liu M., Sun X., Yang Y., Hao M., Sheng S., Sun Y., Zhang H. (2014). Huaier polysaccharides suppresses hepatocarcinoma MHCC97-H cell metastasis via inactivation of EMT and AEG-1 pathway. Int. J. Biol. Macromol..

[105] Li T., Xu W.S., Wu G.S., Chen X.P., Wang Y.T., Lu J.J. (2014). Platycodin D induces apoptosis, and inhibits adhesion, migration and invasion in HepG2 hepatocellular carcinoma cells. Asian Pac. J. Cancer Prev..

[106] Yamazaki T., Tokiwa T. (2010). Isofraxidin, a coumarin component from *Acanthopanax senticosus*, inhibits matrix metalloproteinase-7 expression and cell invasion of human hepatoma cells. Biol. Pharm. Bull..

[107] Huang C.S., Lyu S.C., Chen J.Y., Hu M.L. (2012). The anti-metastatic efficacy of β-ionone and the possible mechanisms of action in human hepatocarcinoma SK-Hep-1 cells. Br. J. Nutr..

[108] Yeh M.H., Kao S.T., Hung C.M., Liu C.J., Lee K.H., Yeh C.C. (2009). Hesperidin inhibited acetaldehyde-induced matrix metalloproteinase-9 gene expression in human hepatocellular carcinoma cells. Toxicol. Lett..

[109] Liu X., Yang Y., Zhang X., Xu S., He S., Huang W., Roberts M.S. (2010). Compound *Astragalus* and *Salvia*
*miltiorrhiza* extract inhibits cell invasion by modulating transforming growth factor-β/SMAD in HepG2 cell. J. Gastroenterol. Hepatol..

[110] Tian Q.E., Li H.D., Yan M., Cai H.L., Tan Q.Y., Zhang W.Y. (2012). Astragalus polysaccharides can regulate cytokine and P-glycoprotein expression in H22 tumor-bearing mice. World J. Gastroenterol..

[111] Wang X.B., Wang S.S., Zhang Q.F., Liu M., Li H.L., Liu Y., Wang J.N., Zheng F., Guo L.Y., Xiang J.Z. (2010). Inhibition of tetramethylpyrazine on P-gp, MRP2, MRP3 and MRP5 in multidrug resistant human hepatocellular carcinoma cells. Oncol. Rep..

[112] Liang G., Tang A., Lin X., Li L., Zhang S., Huang Z., Tang H., Li Q.Q. (2010). Green tea catechins augment the antitumor activity of doxorubicin in an *in vivo* mouse model for chemoresistant liver cancer. Int. J. Oncol..

[113] Yue G.G., Kin-Ming Lee J., Cheng L., Chung-Lap Chan B., Jiang L., Fung K.P., Leung P.C., Bik-San Lau C. (2012). Reversal of P-glycoprotein-mediated multidrug resistance in human hepatoma cells by hedyotiscone A, a compound isolated from *Hedyotis corymbosa*. Xenobiotica.

[114] Cheung J.Y., Ong R.C., Suen Y.K., Ooi V., Wong H.N., Mak T.C., Fung K.P., Yu B., Kong S.K. (2005). Polyphyllin D is a potent apoptosis inducer in drug-resistant HepG2 cells. Cancer Lett..

[115] Yang L., Liu X., Lu Z., Yuet-Wa Chan J., Zhou L., Fung K.P., Wu P., Wu S. (2010). Ursolic acid induces doxorubicin-resistant HepG2 cell death via the release of apoptosis-inducing factor. Cancer Lett..

[116] Wong V.K., Chiu P., Chung S.S., Chow L.M., Zhao Y.Z., Yang B.B., Ko B.C. (2005). Pseudolaric acid B, a novel microtubule-destabilizing agent that circumvents multidrug resistance phenotype and exhibits antitumor activity *in vivo*. Clin. Cancer Res..

[117] Li X., Zeng X., Sun J., Li H., Wu P., Fung K.P., Liu F. (2014). Imperatorin induces Mcl-1 degradation to cooperatively trigger Bax translocation and Bak activation to suppress drug-resistant human hepatoma. Cancer Lett..

[118] Cheng C.Y., Su C.C. (2010). Tanshinone IIA inhibits Hep-J5 cells by increasing calreticulin, caspase 12 and GADD153 protein expression. Int. J. Mol. Med..

[119] Wang H.C., Chung P.J., Wu C.H., Lan K.P., Yang M.Y., Wang C.J. (2011). *Solanum nigrum* L. polyphenolic extract inhibits hepatocarcinoma cell growth by inducing G2/M phase arrest and apoptosis. J. Sci. Food Agric..

[120] Lin S.B., Li C.H., Lee S.S., Kan L.S. (2003). Triterpene-enriched extracts from *Ganoderma lucidum* inhibit growth of hepatoma cells via suppressing protein kinase C, activating mitogen-activated protein kinases and G2-phase cell cycle arrest. Life Sci..

[121] Deng S., Hu B., An H.M. (2012). Traditional Chinese Medicinal Syndromes and treatment in colorectal cancer. J. Cancer Ther..

[122] Wang Z., Han W., Sui X., Fang Y., Pan H. (2014). Autophagy: A novel therapeutic target for hepatocarcinoma (Review). Oncol. Lett..

[123] Sui X., Chen R., Wang Z., Huang Z., Kong N., Zhang M., Han W., Lou F., Yang J., Zhang Q. (2013). Autophagy and chemotherapy resistance: A promising therapeutic target for cancer treatment. Cell Death Dis..

[124] Xie B.S., Zhao H.C., Yao S.K., Zhuo D.X., Jin B., Lv D.C., Wu C.L., Ma D.L., Gao C., Shu X.M. (2011). Autophagy inhibition enhances etoposide-induced cell death in human hepatoma G2 cells. Int. J. Mol. Med..

[125] Shi Y.H., Ding Z.B., Zhou J., Hui B., Shi G.M., Ke A.W., Wang X.Y., Dai Z., Peng Y.F., Gu C.Y. (2011). Targeting autophagy enhances sorafenib lethality for hepatocellular carcinoma via ER stress-related apoptosis. Autophagy.

[126] Muehlich S., Gudermann T. (2013). Pro-senescence therapy for hepatocellular carcinoma. Aging (Albany NY).

[127] Hu B., Du Q., Deng S., An H.M., Pan C.F., Shen K.P., Xu L., Wei M.M., Wang S.S. (2014). *Ligustrum lucidum* Ait. fruit extract induces apoptosis and cell senescence in human hepatocellular carcinoma cells through upregulation of p21. Oncol. Rep..

[128] Hu B., An H.M., Shen K.P., Xu L., Du Q., Deng S., Wu Y. (2012). Liver Yin deficiency tonifying herbal extract induces apoptosis and cell senescence in Bel-7402 human hepatocarcinoma cells. Exp. Ther. Med..

[129] Cao L., Han L., Zhang Z., Li J., Qu Z., Du J., Liang X., Liu Y., Liu H., Shi Y. (2009). Involvement of anoikis-resistance in the metastasis of hepatoma cells. Exp. Cell Res..

[130] Hu B., An H.M., Shen K.P., Song H.Y., Deng S. (2012). *Polygonum cuspidatum* extract induces Anoikis in hepatocarcinoma cells associated with generation of reactive oxygen species and downregulation of focal adhesion kinase. Evid. Based Complement. Altern. Med..

[131] Hu B., An H.M., Shen K.P., Xu L., Du Q., Deng S., Wu Y. (2011). Modified Yi Guan Jian, a Chinese herbal formula, induces anoikis in Bel-7402 human hepatocarcinoma cells *in vitro*. Oncol. Rep..

[132] van Zijl F., Zulehner G., Petz M., Schneller D., Kornauth C., Hau M., Machat G., Grubinger M., Huber H., Mikulits W. (2009). Epithelial-mesenchymal transition in hepatocellular carcinoma. Future Oncol..

[133] Shang Y., Cai X., Fan D. (2013). Roles of epithelial-mesenchymal transition in cancer drug resistance. Curr. Cancer Drug Targets.

[134] Xiong W., Ren Z.G., Qiu S.J., Sun H.C., Wang L., Liu B.B., Li Q.S., Zhang W., Zhu X.D., Liu L. (2010). Residual hepatocellular carcinoma after oxaliplatin treatment has increased metastatic potential in a nude mouse model and is attenuated by Songyou Yin. BMC Cancer.

[135] Chen D., Yao W.J., Zhang X.L., Han X.Q., Qu X.Y., Ka W.B., Sun D.G., Wu X.Z., Wen Z.Y. (2010). Effects of Gekko sulfated polysaccharide-protein complex on human hepatoma SMMC-7721 cells: Inhibition of proliferation and migration. J. Ethnopharmacol..

[136] Yau T., Chan P., Epstein R., Poon R.T. (2008). Evolution of systemic therapy of advanced hepatocellular carcinoma. World J. Gastroenterol..

[137] Huang C., Xu D., Xia Q., Wang P., Rong C., Su Y. (2012). Reversal of P-glycoprotein-mediated multidrug resistance of human hepatic cancer cells by Astragaloside II. J. Pharm. Pharmacol..

[138] He Y.L., Ying Y., Xu Y.L., Su J.F., Luo H., Wang H.F. (2005). Effects of *Lycium barbarum* polysaccharide on tumor microenvironment T-lymphocyte subsets and dendritic cells in H22-bearing mice. J. Chin. Integr. Med..

[139] Chen J., Chen J., Wang X., Liu C. (2013). Anti-tumour effects of polysaccharides isolated from *Artemisia annua* L by inducing cell apoptosis and immunomodulatory anti-hepatoma effects of polysaccharides. Afr. J. Tradit. Complement. Altern. Med..

[140] Shu G., Yang T., Wang C., Su H., Xiang M. (2013). Gastrodin stimulates anticancer immune response and represses transplanted H22 hepatic ascitic tumor cell growth: Involvement of NF-κB signaling activation in CD4+ T cells. Toxicol. Appl. Pharmacol..

[141] Long S., GuangZhi Y., BaoJie G., Wei X., YanYong H., YingLi W., Yang Z., LiHua L. (2012). Shikonin derivatives protect immune organs from damage and promote immune responses *in vivo* in tumour-bearing mice. Phytother. Res..

[142] Chen Y.T., Lin M.A., Cheng D.Q., Shi Z.J., Zhu J.L., Wu J. (2009). Effect of proteins extracted from mycelia of *Omphalia lapidescens* on inhibiting H, liver cancer in mice and regulating immune function. J. Chin. Med. Mater..

[143] Ge G.F., Yu C.H., Yu B., Shen Z.H., Zhang D.L., Wu Q.F. (2012). Antitumor effects and chemical compositions of *Eupolyphaga sinensis* Walker ethanol extract. J. Ethnopharmacol..

[144] Chen J.R., Li E.Q., Dai C.Q., Yu B., Wu X.L., Huang C.R., Chen X.Y. (2012). The inducible effect of LBP on maturation of dendritic cells and the related immune signaling pathways in hepatocellular carcinoma (HCC). Curr. Drug Deliv..

[145] Chen D., Zhang X., Du Y., Jia B., Ka W., Sun D., Yao W., Wen Z. (2012). Effects of Gekko sulfated polysaccharide-protein complex on the defective biorheological characters of dendritic cells under tumor microenvironment. Cell Biochem. Biophys..

[146] Li Q., Bao J.M., Li X.L., Zhang T., Shen X.H. (2012). Inhibiting effect of Astragalus polysaccharides on the functions of CD4+CD25 high Treg cells in the tumor microenvironment of human hepatocellular carcinoma. Chin. Med. J. (Engl.).

[147] He X., Li X., Liu B., Xu L., Zhao H., Lu A. (2011). Down-regulation of Treg cells and up-regulation of TH1/TH2 cytokine ratio were induced by polysaccharide from *Radix Glycyrrhizae* in H22 hepatocarcinoma bearing mice. Molecules.

[148] Zhang S.X., Zhu C., Ba Y., Chen D., Zhou X.L., Cao R., Wang L.P., Ren Y., Wu X.Z. (2012). Gekko-sulfated glycopeptide inhibits tumor angiogenesis by targeting basic fibroblast growth factor. J. Biol. Chem..

[149] Mu P., Gao X., Jia Z.J., Zheng R.L. (2008). Natural antioxidant pedicularioside G inhibits angiogenesis and tumourigenesis *in vitro* and *in vivo*. Basic Clin. Pharmacol. Toxicol..

[150] Wang J., Zheng X., Zeng G., Zhou Y., Yuan H. (2014). Purified vitexin compound 1 inhibits growth and angiogenesis through activation of FOXO3a by inactivation of Akt in hepatocellular carcinoma. Int. J. Mol. Med..

[151] Zhang H., Yang R. (2014). Resveratrol inhibits VEGF gene expression and proliferation of hepatocarcinoma cells. Hepatogastroenterology.

[152] Zhang Q., Tang X., Lu Q.Y., Zhang Z.F., Brown J., le A.D. (2005). Resveratrol inhibits hypoxia-induced accumulation of hypoxia-inducible factor-1α and VEGF expression in human tongue squamous cell carcinoma and hepatoma cells. Mol. Cancer Ther..

[153] Chen T., Fu Y.L., Gong Z.P., Deng L.R., Hu Y.Q. (2010). Studies on the anti-angiogenic mechanism of the formula of Chinese medicine active ingredients combined with small dose cisplatin in mice of hepatocellular carcinoma. Chin. J. Exp. Tradit. Med. Formul..

[154] Deng S., Hu B., An H.M., Du Q., Xu L., Shen K.P., Shi X.F., Wei M.M., Wu Y. (2013). Teng-Long-Bu-Zhong-Tang, a Chinese herbal formula, enhances anticancer effects of 5-Fluorouracil in CT26 colon carcinoma. BMC Complement. Altern. Med..

[155] Bishayee A., Darvesh A.S. (2012). Angiogenesis in hepatocellular carcinoma: A potential target for chemoprevention and therapy. Curr. Cancer Drug Targets.

[156] Song P., Wang X.M., Xie S. (2006). Experimental study on mechanisms of lyophilized powder of fresh gekko Chinenis in inhibiting H22 hepatocarcinoma angiogenesis. Chin. J. Integr. Tradit. West. Med..

[157] Hu B., Du Q., Shen K.P., Xu L. (2012). Principles and scientific basis of Traditional Chinese Medicine in cancer treatment. J. Bioanal. Biomed..

[158] Du Q., Hu B., An H.M., Shen K.P., Xu L., Deng S., Wei M.M. (2013). Synergistic anticancer effects of curcumin and resveratrol in Hepa1–6 hepatocellular carcinoma cells. Oncol. Rep..

[159] Chen T., Li D., Fu Y.L., Hu W. (2008). Screening of QHF formula for effective ingredients from Chinese herbs and its anti-hepatic cell cancer effect in combination with chemotherapy. Chin. Med. J. (Engl.).

[160] Wu Y.Y., Wan L.H., Zheng X.W., Shao Z.J., Chen J., Chen X.J., Liu L.T., Kuang W.J., Tan X.S., Zhou L.M. (2012). Inhibitory effects of β,β-dimethylacrylshikonin on hepatocellular carcinoma *in vitro* and *in vivo*. Phytother. Res..

[161] Xiong W., Luo G., Zhou L., Zeng Y., Yang W. (2009). *In vitro* and *in vivo* antitumor effects of acetylshikonin isolated from *Arnebia euchroma* (Royle) Johnst (Ruanzicao) cell suspension cultures. Chin. Med..

[162] Wu Y.Y., Zhu L., Ma X.Y., Shao Z.J., Chen J., Chen X.J., Wan L.H., Zhou L.M. (2011). The anti-proliferation effect of Aikete injection on hepatocellular carcinoma *in vitro* and *in vivo*. Pharm. Biol..

